# *Bifidobacterium bifidum* and *Lactobacillus paracasei* alleviate sarcopenia and cognitive impairment in aged mice by regulating gut microbiota-mediated AKT, NF-κB, and FOXO3a signaling pathways

**DOI:** 10.1186/s12979-023-00381-5

**Published:** 2023-10-23

**Authors:** Ji-Su Baek, Yoon-Jung Shin, Xiaoyang Ma, Hee-Seo Park, Yun-Ha Hwang, Dong-Hyun Kim

**Affiliations:** 1 Neurobiota Research Center, College of Pharmacy, Kyung Hee University, Dongdaemun-gu, Seoul, 02447 Korea; 2 DongWha Pharm Research Institute, Yongin-shi, Gyeonggi, 17084 Korea; 3 PBLbiolab, Seongbuk-gu, Seoul, 02823 Korea

**Keywords:** Sarcopenia, Muscle atrophy, Protein synthesis, Gut microbiota, *Bifidobacterium bifidum*, *Lactobacillus paracasei*

## Abstract

Sarcopenia is closely associated with gut dysbiosis. Probiotics alleviate gut dysbiosis. Therefore, we selected probiotics *Lactobacillus paracasei* P62 (Lp) and *Bifidobacterium bifidum* P61 (Bb), which suppressed muscle RING-finger protein-1 (MuRF1) expression and NF-κB activation in C2C12 cells, and examined their effects on muscle mass loss and dysfunction in aged mice. Oral administration of Lp, Bb, or their mix (LB) increased grip strength and treadmill running distance and time. They significantly increased muscle weight in aged mice. They also increased AKT activation, PGC1α, SIRT1, and myosin heavy chain (MyHC) expression, MyHC-positive cell population, and cell size in the gastrocnemius (GA) muscle, while FOXO3a and NF-κB activation, MuRF1, muscle atrophy F-box, and p16 expression, and NF-κB^+^CD11c^+^ cell population decreased. Furthermore, they reduced cognitive impairment-like behavior, IL-6 expression, FOXO3a activation, and NF-κB-positive cell population in the hippocampus, GA, and colon, while hippocampal brain-derived neurotropic factor expression increased. They shifted gut microbiota composition in aged mice: they increased *Akkermansiaceae* and *Bacteroidaceae* populations, which were positively correlated with total muscle weight and MyHC expression, and decreased *Odoribacteraceae* and *Deferribacteriaceae* populations, which were positively correlated with MuRF1 and IL-6 expression. LB alleviated sarcopenia- and cognitive impairment-like symptoms more potently than Lp or Bb alone. Based on these findings, probiotics, particularly Lp, Bb, and LB, can alleviate aging-dependent sarcopenia and cognitive impairment by regulating gut microbiota-mediated AKT, NF-κB, and/or FOXO3a signaling pathways.

**Supplementary Information:**

The online version contains supplementary material available at 10.1186/s12979-023-00381-5.

## Introduction

Sarcopenia is a progressive disorder characterized by significant loss of skeletal muscle mass and strength, and is observed in various diseases such as cancer, cirrhosis, and chronic obstructive pulmonary disease [1, 2]. Aging is the main risk factor for sarcopenia with Alzheimer’s disease [3]. Inflammation, oxidative stress, and mitochondrial dysfunction induced by stressors such as aging and pathogens cause an imbalance of protein synthesis and protein degradation in skeletal muscles, which prevents myoblast activation, proliferation, and differentiation, resulting in sarcopenia [4, 5]. Skeletal muscle mass and function in mice are reported to be regulated by gut microbiota and their metabolites, which cause muscle atrophy in germ-free mice [6]. Fecal microbiota transplantation from young mice rejuvenates physical fitness with muscle thickness in the transplanted aged mice [7]. Fecal microbiota transplantation from pigs with myostatin deletion alleviates muscle atrophy in pigs [8]. Therefore, sarcopenia may be closely associated with gut microbiota.

Probiotics exhibit anti-inflammatory, anti-depressive, and cognitive impairment-ameliorating effects [9, 10]. In addition, Chen et al. reported that *Lactobacillus casei* Shirota, which was isolated from a fermented food, had significant anti-inflammatory effects in mice and human studies and induced muscles in aged mice [11]. Lee et al. reported that *Lactobacillus plantarum* HY7715 isolated from kimchi increased skeletal muscle mass and function in aged mice, resulting in the amelioration of sarcopenia [12]. Chen et al. reported that *Lactobacillus paracasei* PS23 isolated from human feces attenuated muscle loss in senescence-accelerated mouse prone-8 (SAMP8) mice by ensuring mitochondrial function [13]. Munukka et al. reported that *Faecalibacterium prausnitzii* reduced systemic inflammation and increased muscle mass in high‐fat fed mice [14]. A mixed supplement of multiple probiotics also reduces inflammation and muscle atrophy markers [15]. Nevertheless, the action mechanism of sarcopenia-ameliorating probiotics remains elusive.

Therefore, to confirm whether probiotics could alleviate sarcopenia, we first screened muscle RING-finger protein-1 (MuRF1, a muscle atrophy factor) expression-suppressing probiotics *Lactobacillus paracasei* P62 (Lp) *and Bifidobacterium bifidum* P61 (Bb) in dexamethasone- or LPS-treated C2C12 cells and examined their effects in aged mice.

## Methods

### Culture of probiotics

Probiotics including *L. paracasei* P62 (KCCM 13368P) and *B. bifidum* P61 (KCCM 13367P), selected from human gut microbiota collection, were cultured in general media for probiotics such as MRS broth (BD, Sparks, MD), collected by centrifugation, and freeze-dried. For the in vitro study, cells were washed with saline twice and suspended in saline. For the in vivo study, cells were suspended in 1% maltose solution.

### Culture of C2C12 cells

C2C12 cells (Korean Type Culture Collection, Seoul, Korea) were cultured in DMEM medium containing 10% fetal bovine serum, 1% antibiotic–antimycotic solution, and 3.7 g/L NaHCO_3_ at 37^o^C in a 5% CO_2_/95% air humidified incubator [16]. Confluent myoblasts (80%) were differentiated in DMEM medium containing 2% horse serum for 4 days. Probiotics (1 × 10^4^ cells/mL) or creatine (Cr, 5 mM, C0780, Sigma) were treated 10 h after treatment with dexamethasone (10 µM, D4902, Sigma) or lipopolysaccharide (LPS, 100 ng/mL) in differentiated C2C12 cells (1 × 10^5^ cells/mL).

### Animals

Aged C57BL/6 mice (male, 18 months old) and young C57BL/6 mice (male, 12-weeks old) were purchased from Orientbio Co. ltd., maintained in a controlled room with water and food ad libitum, and acclimatized for 7 days before the experimental use. All animal experiments were approved by the Committee for the Care and Use of Laboratory Animals in Kyung Hee University (IACUC No, KHUASP(SE)-22-567) and were ethically carried in accordance of the Guideline of the University for Laboratory Animals Care and Use.

We randomly divided mice into six groups (Yg, Vh, Lp, Bb, LB, and Cr). Each group consisted of 6 mice. Test agents (Yg, vehicle in young mice; Vh, vehicle in aged mice; Lp, 1 × 10^9^ CFU/mouse of *Lactobacillus paracasei* P62 in aged mice; Bb, 1 × 10^9^ CFU/mouse of *Bifidobacterium bifidum* P61 in aged mice; LB, 1 × 10^9^ CFU/mouse of *Bifidobacterium bifidum* P61 and *Lactobacillus paracasei* P62 (1:4) mix in aged mice; Cr, 75 mg/kg of creatine in aged mice) were orally gavaged once a day for 8 weeks (6 days per week). Physical performance was measured using grip strength and treadmill exercise tests 20 h after the final treatment with test agents. Cognitive function-like behaviors were measured 20 h after the physical performance tests. Mice were euthanized by the exposure to CO_2_ in the chamber and then sacrificed by cervical dislocation. Sera, brains, colons, and feces were then collected and stored at – 80 °C for biomarker assays.

### Physical performance test

For the measurement of grip strength, mice were placed with their all limbs on the grid of a grip strength meter and grip strength was measured immediately before mice fell from the bar [17].

For the measurement of treadmill running time and distance, mice were first adapted for 1 week to become familiar with treadmill before treadmill exercise. Total running time and distance were measured at speed of 23 m/min for 30 min, as previously reported [17].

### Cognitive function-like behaviors

The cognitive behavior (Y-maze) task was measured in a three-arm (120^o^) horizontal maze consisted of 40-cm-long and 3-cm-wide with 12-cm-high walls, as previously reported [18].

### Enzyme-linked immunosorbent assay (ELISA) and immunoblotting

Muscle, colon, and brain tissues and cells were homogenized in the RIPA lysis buffer containing 1% phosphatase inhibitor cocktail and 1% protease inhibitor cocktail (RPP) on ice and centrifuged at 15,000 *g* at 4 °C for 15 min. The biomarker levels were assayed, as previously reported [19].

For the assay of cytokines, the homogenate supernatants were transferred in 96-well plates and assayed using ELISA kits (R&D system, Minneapolis, MN).

For the immunoblotting analysis, the homogenate supernatants of tissues and cells were subjected to sodium dodecyl sulfate-polyacrylamide gel electrophoresis and transferred to polyvinylidene fluoride. Proteins were probed with antibodies, detected with horseradish peroxidase-conjugated secondary antibodies, and visualized with enhanced chemiluminescence detection kit.

The used antibodies are follows: phospho-Akt (Ser473) (193H12, Cell Signaling, Danvers, MA), Akt (11E7) (4685, Cell Signaling), muscle atrophy F-box gene (MAFbx, F-9, sc-166,806, Santa Cruz Biotechnology, Santa Cruz, CA), MuRF1(C-11) (sc-398,608, Santa Cruz Biotechnology), phospho-mTOR (ser2448) (2971, Cell Signaling), mammalian target of rapamycin (mTOR, 7C10, 2983,Cell Signaling), p-NF-κB-p65 (S536) (93H1, Cell Signaling), NF-κB-p65 (D14E12) (8242, Cell Signaling), myosin heavy chain (MyHc, B-5, sc-376,157, Santa Cruz Biotechnology), p16INK4A (E5F3Y, Cell signaling), p-FOXO3a (ser253, Cell Signaling), FOXO3a (75D8, Cell Signaling), β-actin (sc-47,778, Santa Cruz Biotechnology), and peroxisome proliferator-activated receptor gamma coactivator (PGC)1α (ab191838, Abcam).

### Quantitative real-time polymerase chain reaction (qPCR) analysis

Total RNA was purified from C2C12 cells and GA muscles using Qiagen RNeasy mini kit and Qiagen RNeasy Fibrous Tissue mini kit, respectively. The isolated RNA (2 µg) was reverse-transcribed using a PrimeScript cDNA synthesis kit (Takara, Shiga, Japan). qPCR was performed using the Rotor-Gene Q 5plex Platform (Qiagen) with TB Green Premix Ex Taq II (Takara). Primer sequences were described in Supplement (Table S1).

#### Mitochondrial DNA (mtDNA) analysis

Total DNA was extracted from C2C12 cells and GA muscle using Genomic DNeasy kit (Qiagen). The mtDNA copy number was measured via qRT-PCR. Primer sequences were described in Supplement (Table S1).

### Hematoxylin & eosin staining

Mice were transcardiacally perfused with paraformaldehyde. Gastrocnemius (GA) muscles were cut into 5 μm sections and stained with hematoxylin and eosin (H&E) [12].

### Immunofluorescence staining

Hypothalamus, GA muscle, and colon tissues were collected from mice transcardiacally perfused with paraformaldehyde, sectioned, incubated with primary antibodies (for brain-derived neurotropic factor [BDNF], NeuN, NF-κB, Iba1, and/or CD11c) for 12 h, then treated with secondary antibodies conjugated with Alexa Fluor 594 or Alexa Fluor 488, and observed using a confocal microscope, as previously reported [20].

### Microbiota analysis

Fecal microbiota genomic DNAs were extracted using a QIAamp DNA stool mini kit. 16 S rRNA genes were amplified and sequenced, as previously reported [21]. Sequenced data were deposited in the NCBI (PRJNA1011499).

### Statistics

Data are expressed as mean ± SD using GraphPad Prism. The significance (p < 0.05) for all data except muscle weights was analyzed using one-way ANOVA followed by Dunnett’s multiple range test. The significance for muscle weights was analyzed using one-way ANOVA followed by Fisher’s LSD test. The correlation between gut microbiota and total muscle weight, GA weight, tumor necrosis factor (TNF)-α/interleukin (IL)-10, IL-6/IL-10, MuRF1, or MyHC expression level was analyzed using Spearman correlation coefficient.

## Results

### Lp and Bb suppressed dexamethasone-induced MuRF1 expression in C2C12 cells

**Fig. 1:**
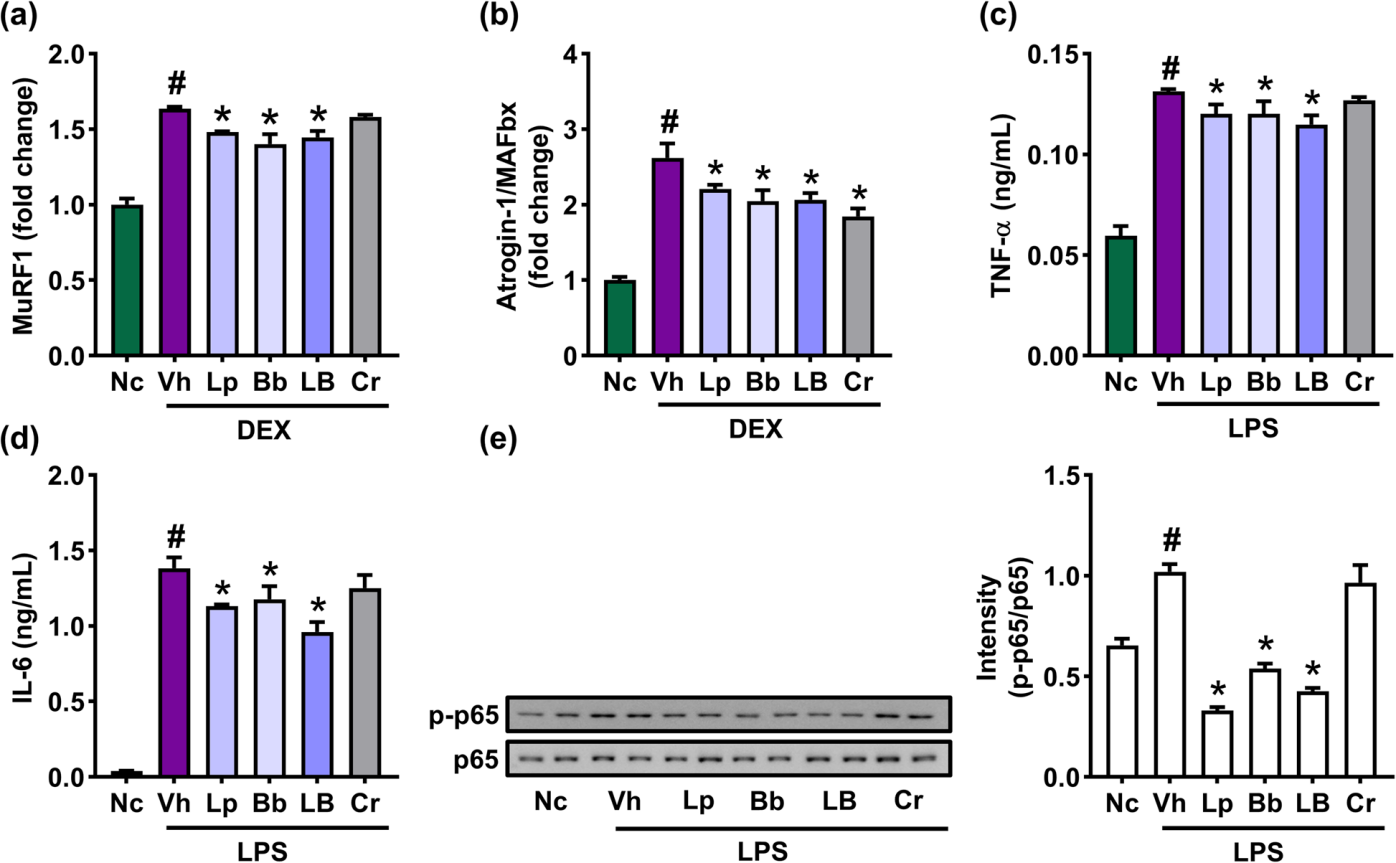
Effects of Bb and Lp on dexamethasone-induced MuRF1 and Atrogin-1/MAFbx expression and LPS-induced TNF-α and IL-6 expression and NF-κB activation in C2C12 cells. Effects on MuRF1 (**a**) and Atrogin-1/MAFbx expression (**b**). Effects on TNF-α (**c**) and IL-6 expression (**d**) and NF-κB activation (**e**). C2C12 cells (1 × 10^5^ cells/mL) were treated with dexamethasone (10 µM) or LPS (100 ng/mL) in the absence or presence of Bb, Lp, or LB (1 × 10^4^ colon-forming units [CFUs] /mL and Cr [5 mM]). Data are indicated as mean ± SD (n = 4). ^#^*p* < 0.05 vs. NC. ^*^p < 0.05 vs. group treated with vehicle with dexamethasone or LPS

First, we screened MuRF1 expression-suppressing probiotics in the lactic acid bacteria collection isolated from human fecal bacteria using dexamethasone-treated C2C12 cells (Fig. 1). Dexamethasone treatment significantly induced MuRF1 expression. However, of tested bifidobacteria and lactobacilli, P61 and P62 significantly suppressed dexamethasone-induced MuRF1 expression. They also suppressed dexamethasone-induced MAFbx/atrogin-1 expression. They furthermore significantly suppressed LPS-induced tumor necrosis factor (TNF)-α and interleukin (IL)-6 expression and NF-κB activation in C2C12 cells (Fig. 1, Supplement Figure S1). When P61 and P62 were mixed (4:1, 1:1, and 1:4), the [1:4] mix most potently suppressed IL-6 expression in LPS-stimulated C2C12 cells (Supplement Figure S2). P61 and P62 were *Bifidobacterium bifidum* and *Lactobacillus paracasei*, respectively, based on the results of Gram staining, API kit assay, and whole genome analyses. The genome sequences of P61 and P62 showed the highest phylogenetic similarity to *B. bifidum* JCM1255 (98.8%) and *L. paracasei* subsp. *paracasei* JCM 8130 (98.6%), respectively, using OrthoANI (Supplement Tables S2).

### Effects of Lp and Bb on the skeletal muscle strength and mass in aged mice

**Fig. 2:**
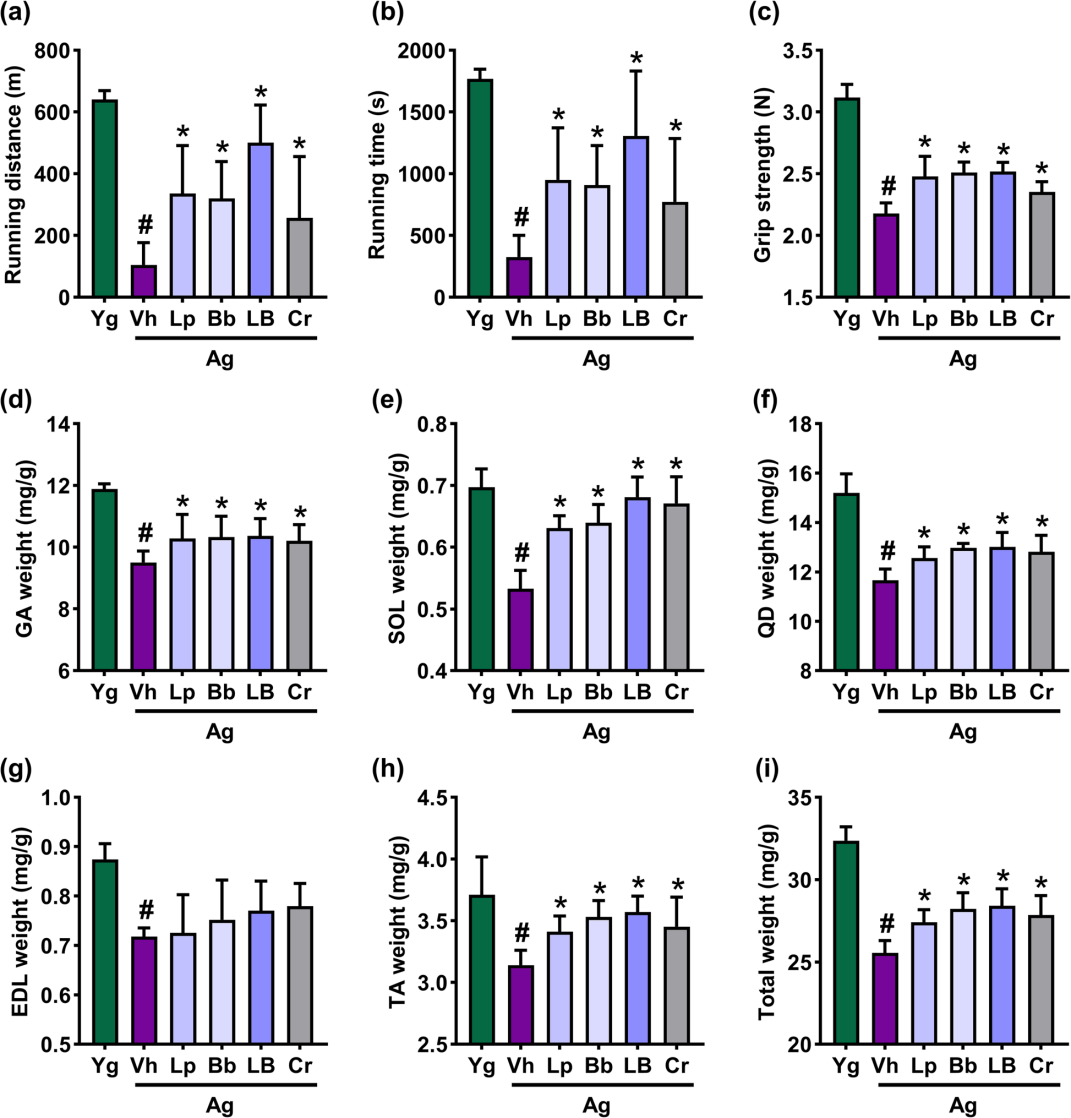
Effects of Bb, Lp, and LB on the skeletal muscle strength and mass in aged mice. Effects on treadmill running distance (**a**) and time (**b**) and grip strength (**c**). Effects on the weights of gastrocnemius (GA, **d**), soleus (SOL, **e**), quadriceps femoris (QD, **f**), extensor digitorum longus (EDL, **g**), tibialis anterior (TA, **h**), and total muscles (**i**). Bb, Lp, and LB (1 × 10^9^ CFU/mouse/day) and Cr (75 mg/kg) were orally gavaged once a day (6 days in one week) for 8 weeks. Data are indicated as mean ± SD (*n* = 6). ^#^*p* < 0.05 vs. Yg. ^*^p < 0.05 vs. Vh/Ag

To confirm whether Lp and Bb could alleviate sarcopenia, we examined their effects on the skeletal muscle mass and strength in aged mice (Fig. 2). The bodyweight of aged mice slowly increased for 8 weeks (Supplement Figure S3). There was no significant difference in food intake and bodyweight gain between aged mice treated with Lp, Bb, LB (their [4:1] mix), creatine (Cr), and vehicle (Vh). First, we examined their effects on the skeletal muscle strength in a treadmill exhaustion test and grip strength tests. The treadmill distance was significantly shorter in aged mice than that in young mice. However, oral administration of LB most potently increased the treadmill distance and running time in aged mice, followed by Lp, Bb, and Cr. In an all-limb test, Lp, Bb, and LB increased grip strength 1.14-, 1.15-. and 1.16-fold, respectively, compared with those treated with vehicle. Lp, Bb, and LB increased grip strength and treadmill distance and running time more potently than Cr.

Next, we investigated the effects of Lp, Bb, and LB on muscle weight. The gastrocnemius (GA), soleus (SOL), quadriceps femoris (QD), extensor digitorum longus (EDL), and tibialis anterior (TA) muscle weight of aged mice were lower than those of young mice. However, oral administration of Lp, Bb, or LB significantly increased the weight of GA, SOL, QD, EDL, and TA muscles. Of these, LB most potently increased their weight: it increased the weight of GA, SOL, QD, EDL, and TA to 109.1%, 132.4%, 107.3%, 115.4%, and 106.1% of aged mice.

### Effects of Lp and Bb on the AKT signal activation in the skeletal muscle of aged mice

**Fig. 3:**
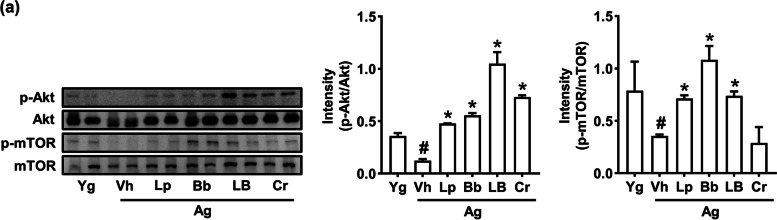
Effects of Bb, Lp, and LB on the AKT and mTOR activation in the skeletal muscle of aged mice. Bb, Lp, and LB (1 × 10^9^ CFU/mouse/day) and Cr (75 mg/kg) were orally gavaged once a day (6 days in one week) for 8 weeks. Data are indicated as mean ± SD (n = 6). ^#^*p* < 0.05 vs. Yg. ^*^p < 0.05 vs. Vh/Ag

To understand the action mechanism of probiotics on muscle weight and strength, we examined the effects of Lp, Bb, and LB on protein synthesis-involved AKT and mTOR activation in the GA muscle in aged mice (Fig. 3, Supplement Figure S4). The activation of AKT and mTOR was lower in the GA of aged mice than in those of young mice. Oral administration of Lp, Bb, or LB increased AKT and mTOR activation in aged mice.

### Effects of Lp and Bb on the myogenesis-related gene expression in the skeletal muscle of aged mice

**Fig. 4:**
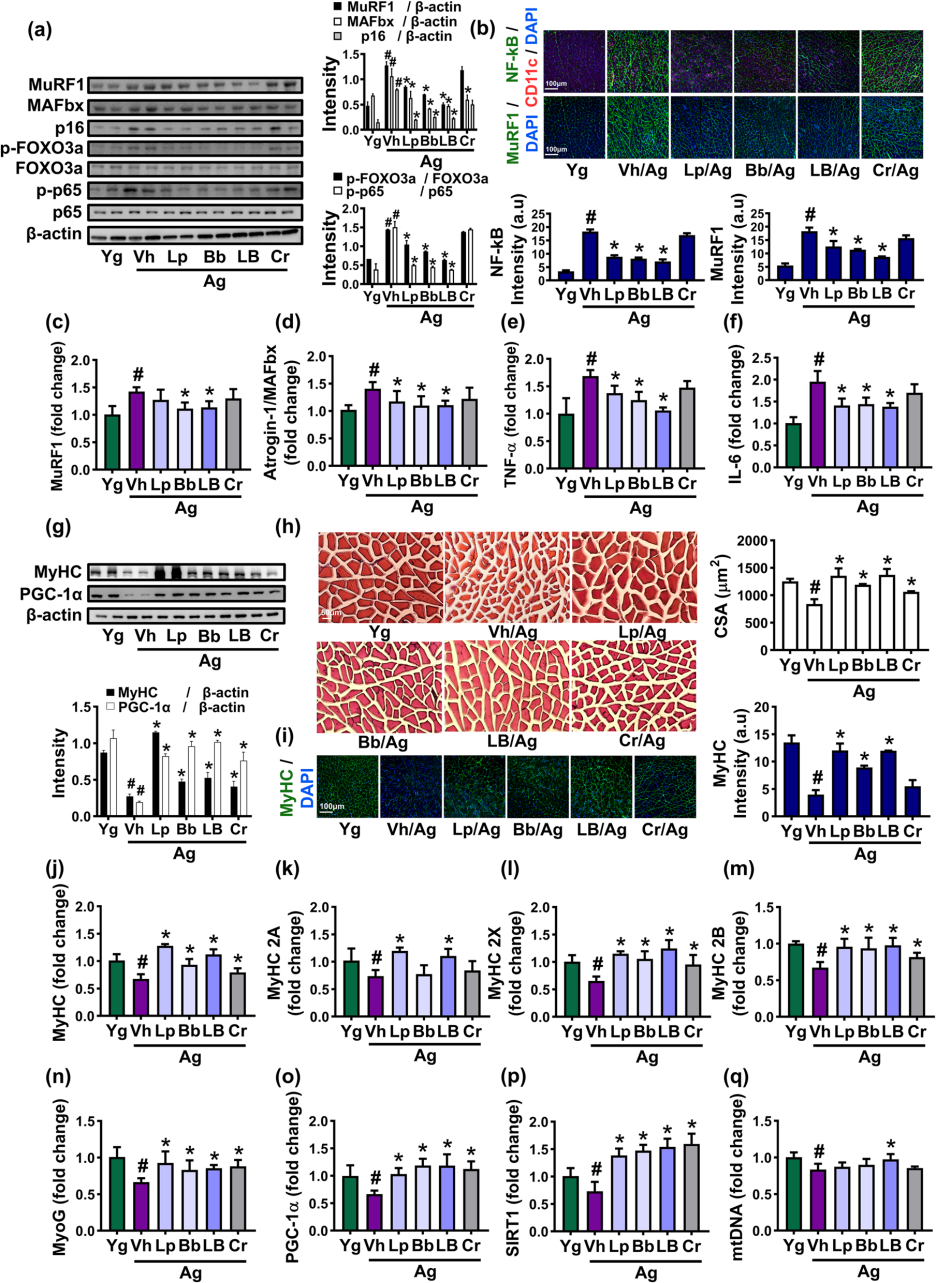
Effects of Bb, Lp, and LB on the myogenesis gene expression in the GA muscle of aged mice. **a** Effects on MuRF-1, MAFbx, p16, p-FOXO3a, FOXO3a, p-p65, p65, and β-actin expression, assessed by immunoblotting. **b** Effects on NF-κB^+^/CD11c^+^ and MuRF1^+^ cell population, assessed by immunofluorescence staining. Effects on MuRF-1 (**c**), MAFbx/Atrogin-1 (**d**), TNF-α (**e**), and IL-6 expression (**f**), assessed by qPCR. Effects on PGC1α, MyHC, and β-action expression (**g**, immunoblotted), GA muscle cell size (**h**, H&E-stained), and MyHC-positive cell population (**i**, stained with immunofluorescence-stained). Effects on MyHC (**j**), MyHC 2 A (**k**), MyHC 2X (**l**), MyHC 2B (**m**), MyoG (**n**), PGC1a (**o**), SIRT1 (**p**), and mtDNA (**q**) expression (**n**), assessed by qPCR. Bb, Lp, and LB (1 × 10^9^ CFU/mouse/day) and Cr (75 mg/kg) were orally gavaged once a day (6 days in one week) for 8 weeks. Data are indicated as mean ± SD (n = 6). ^#^*p* < 0.05 vs. Yg. ^*^p < 0.05 vs. Vh/Ag

FOXO3a and NF-κB are transcription factors that regulates the transcription of mitogenesis-related genes in the muscle [22, 23]. The activation of these factors suppresses myoblast-differentiating myogenesis genes including MyHC and myogenin (MyoG) and induces muscle-degrading atrogenes including MuRF1 and MAFbx. We also found that FOXO3a and NF-κB were potently activated in the GA muscle of aged mice compared to that of young mice (Fig. 4). Therefore, we examined the effects of Lp, Bb, and LB on the myogenesis gene expression in the GA muscle of aged mice (Fig. 4, Supplement Figure S5). Oral administration of Lp, Bb, or LB significantly suppressed FOXO3a and NF-κB activation in aged mice. Furthermore, they decreased MuRF1, MAFbx, and p16 expression, assessed by immunoblotting. In the immunofluorescence staining, they also decreased MuRF^+^ and NF-κB^+^CD11c^+^ cell populations. Furthermore, they suppressed TNF-α and IL-6 expression. However, Cr did not affect their expression.

Oral administration of Lp, Bb, or LB increased MyHC and PGC1α expression, assessed by immunoblotting. They also increased muscle cell size and MyHC-positive cell population. In a qPCR analysis, they decreased MuRF1 and MAFbx expression, while MyHC and MyoG expression increased. Of MyHCs, MyHC2A expression was significantly increased by treatment with Lp or LB, while Bb did not affect its expression. However, Lp, Bb, and LB all increased the expression of MyHC2X, MyH2B, and MyHC more potently than Cr.

Next, we investigated the effects of Lp, Bb, and LB on mitochondrial gene expression-involved PGC1α, SIRT1, and mtDNA expression levels in the GA muscle. PGC1α and SIRT1 expression was lower in aged mice than in young mice. However, oral administration of Lp, Bb, or LB significantly increased PGC1α and SIRT1 expression in aged mice, assessed by qPCR analysis.

### Effects of Lp and Bb on the cognitive function in aged mice

**Fig. 5:**
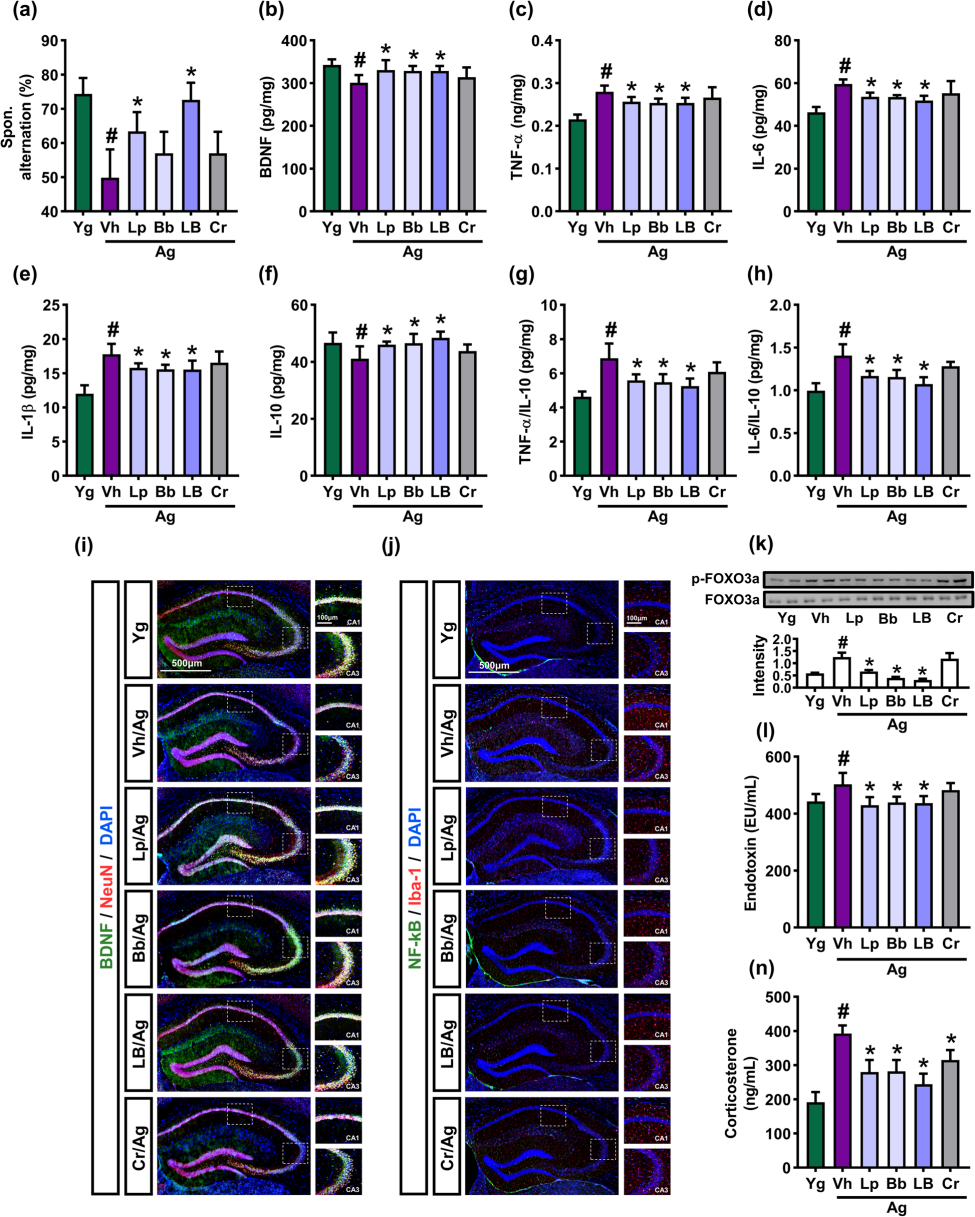
Effects of Bb, Lp, and LB on the cognitive function in aged mice. **a** Effects on spontaneous alternation in the Y-maze task. Effects on hippocampal BDNF (**b**), TNF-α (**c**), IL-6 (**d**), IL-1β (**e**), and IL-10 (**f**) expression, TNF-α to IL-10 expression ratio (**g**), and IL-6 to IL-10 expression ratio (**h**), assessed by qPCR. Effects on BDNF^+^NeuN^+^ (**i**) and NF-κB^+^Iba1^+^ cell populations (**j**). (**k**) Effects on p-FOXO3a and FOXO3a expression, assessed by immunoblotting. Effects on blood endotoxin (**l**) and corticosterone levels (**m**). Bb, Lp, and LB (1 × 10^9^ CFU/mouse/day) and Cr (75 mg/kg) were orally gavaged once a day (6 days in one week) for 8 weeks. Data are indicated as mean ± SD (*n* = 6). ^#^*p* < 0.05 vs. Yg. ^*^p < 0.05 vs. Vh/Ag

Aging induces cognitive decline with systemic inflammation including neuroinflammation [24]. We found that cognitive impairment-like behaviors and TNF-α and IL-6 expression were higher in the hippocampus of aged mice than in those of young mice (Fig. 5, Supplement Figure S6). However, oral administration of Lp, Bb, or LB alleviated cognitive impairment-like behavior in the Y-maze test. They also decreased TNF-α and IL-6 expression, FOXO3a activation, and NF-κB^+^Iba1^+^ cell population, while IL-10 and BDNF expression and BDNF^+^NeuN^+^ cell population increased in the hippocampus. Ageing increases LPS and corticosterone (cortisol) in the blood and LPS levels in the feces [25]. The bacterial endotoxin induces cognitive impairment with systemic inflammation [26]. We also found that LPS and corticosterone levels were higher in the blood of aged mice than in that of young mice. However, oral administration of Lp, Bb, or LB decreased blood LPS and corticosterone levels in aged mice.

### Effects of Bb, Lp, and LB on the gut inflammation and microbiota composition in aged mice

**Fig. 6:**
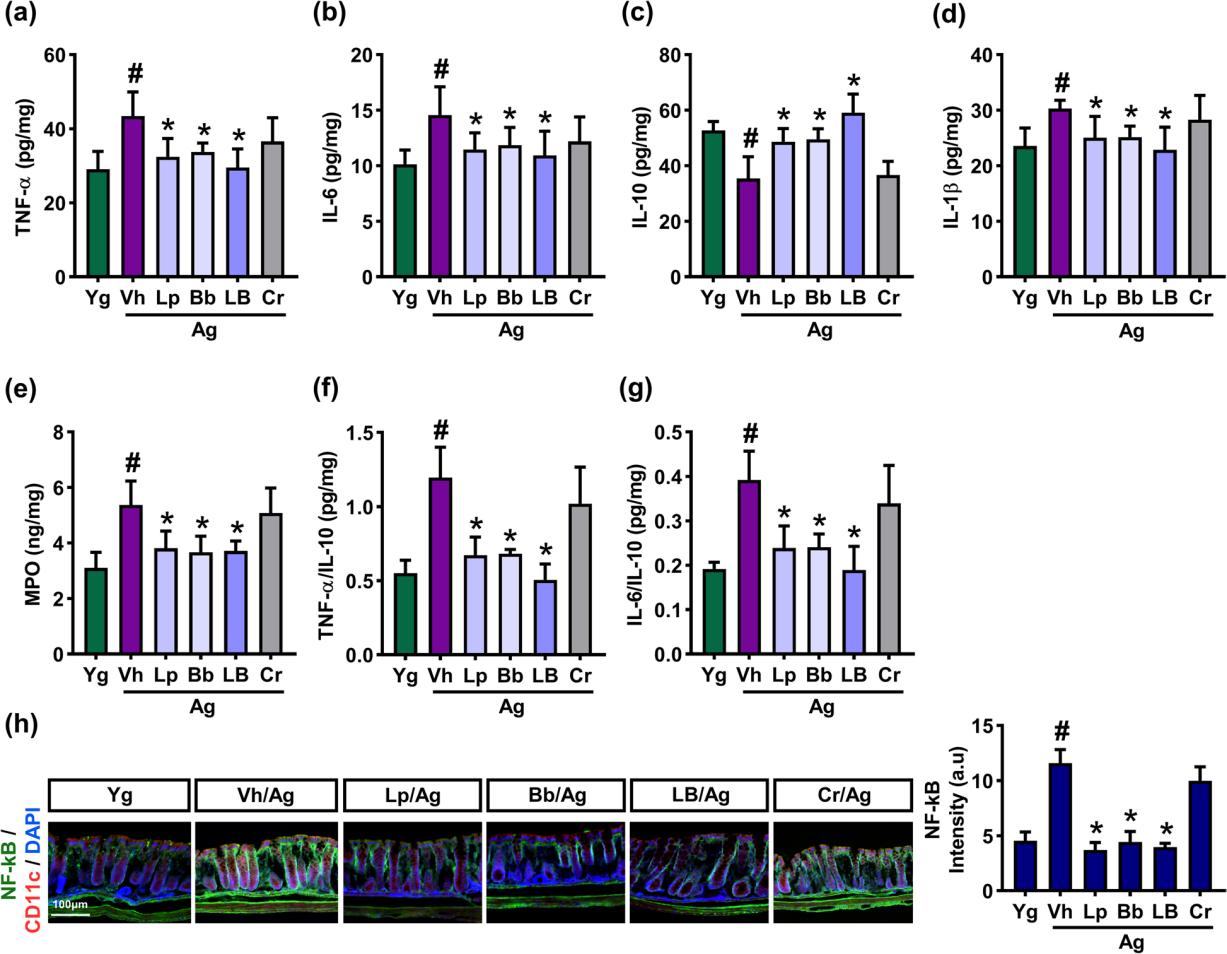
Effects of Bb, Lp, and LB on the gut inflammation in aged mice. Effects on TNF-α (**a**), IL-6 (**b**), IL-10 (**c**), IL-1β (**d**), and myeloperoxidase (MPO) expression (**e**), and TNF-α to IL-10 expression ratio (**f**), and IL-6 to IL-10 expression ratio (**g**), assessed by qPCR. **h** Effects on NF-κB^+^CD11c^+^ cell population. Bb, Lp, and LB (1 × 10^9^ CFU/mouse/day) and Cr (75 mg/kg) were orally gavaged once a day (6 days in one week) for 8 weeks. Data are indicated as mean ± SD (*n* = 6). ^#^*p* < 0.05 vs. Yg. ^*^p < 0.05 vs. Vh/Ag

Ageing increases systemic inflammation including gut inflammation, which induces gut dysbiosis [27]. We found that the expression of inflammatory markers TNF-α, IL-1β, IL-6, and myeloperoxidase was higher in the colon of aged mice than in that of young mice (Fig. 6). Oral administration of Lp, Bb, or LB decreased TNF-α, IL-1β, and IL-6 expression, TNF-α to IL-10 expression ratio, and NF-κB^+^CD11c^+^ cell population in aged mice, while IL-10 expression increased. However, Cr did not affect the expression of inflammatory markers in aged mice.

**Fig. 7:**
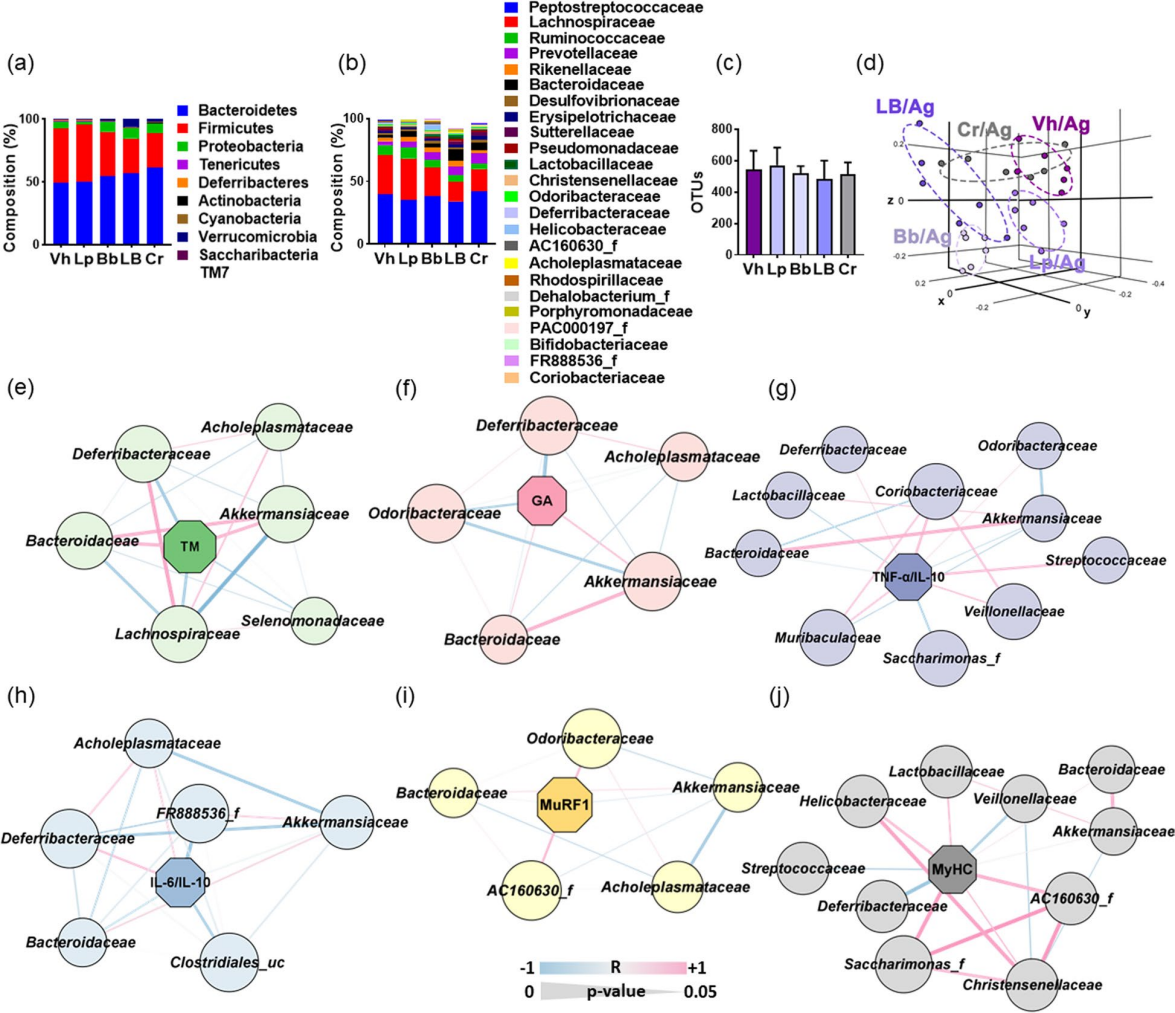
Effects of Bb, Lp, and LB on the gut microbiota composition in aged mice. Effect on the microbiota composition at the phylum (**a**) or family levels (**b**). Effects on OTUs (α-diversity, **c**) and β-diversity (principal coordinate analysis [PCoA] plot based on BrayCurtis) (**d**). The relationship between gut microbiota and total muscle weight (**e**), GA muscle weight (**f**), TNF-α to IL-10 expression ration (**g**), IL-6 to IL-10 expression ratio (**h**), MuRF1 expression (**i**), or MyHC expression (**j**) in the GA, assessed by Spearman coefficient test. Bb, Lp, and LB (1 × 10^9^ CFU/mouse/day) and Cr (75 mg/kg) were orally gavaged once a day (6 days in one week) for 8 weeks. Data are indicated as mean ± SD (*n* = 6). ^*^p < 0.05 vs. Vh/Ag

The gut microbiota composition of aged mice was significantly different to that of young mice (Fig. 7). Although the α-diversity was not significantly different between aged and young mice, the β-diversity significantly different. Oral administration of Lp, Bb, or LB decreased Deferribacteres population in aged mice. They increased Prevotellaceae, Akkermansiaceae, and Bacteroidaceae populations and decreased Odoribacteraceae, Deferribacteraceae, Coriobacteriaceae, and Acholeplasmataceae populations at the family level.

Of these bacteria, Akkermansiaceae and Bacteroidaceae populations, which were increased in aged mice, were positively correlated with total muscle weight, while Lachnospiraceae, Deferribacteraceae, and Selenomonadaceae populations were negatively correlated. GA muscle weight was positively correlated with the populations of Akkermansiaceae and Bacteroidaceae, while Odoribacteraceae, Deferribacteraceae, and Acholeplasmataceae populations were negatively correlated. MuRF1 expression was positively correlated with Odoribacteraceae, AC160630_f, and Acholeplasmataceae populations, while Akkermansiaceae and Bacteroidaceae populations were negatively correlated.

MyHC expression was positively correlated with Saccharimonas_f, SC160630_f, and Lactobacillaceae populations, while Deferribacteriaceae and Veillonellaceae populations were negatively correlated. TNF-α to IL-10 expression ratio was positively correlated with Streptococcaceae, Coriobacteriaceae, Veillonellaceae, and Muribacteriaceae populations, while Saccharimonas_f and Lactobacillaceae populations were negatively correlated. IL-6 to IL-10 expression ratio was positively correlated with Deferribacteraceae and Acholeplasmataceae populations, while FR888536_f, Clostridiales_us, Bacteroidaceae, and Akkermansiaceae populations were negatively correlated.

## Discussion

The muscle mass, quality, and strength are regulated by the orchestrated activation, proliferation, and differentiation of myoblast in the muscle, which are regulated by muscle-specific myogenic regulatory factors such as MyoD, MyoG, and MyHC [21, 28, 29]. MyoD and MyoG are transcription factors involved in regulating the proliferation and differentiation of muscles. MyHC is a major contractile myosin protein expressed in the differentiated muscle. MuRF1 and MAFbx/Atrogin-1 are E3 ubiquitin ligases that regulate ubiquitin-mediated protein degradation in skeletal muscle [30]. In addition, myogenic gene expression (protein synthesis) is increased by the activation of AKT and mTOR, but decreased by the activation of NF-κB [31, 32]. The activation of NF-κB and FOXO3 causes muscle atrophy by inducing MuRF1 and MAFbx and suppressing MyHC expression [33, 34]. Mitochondria-related genes, such as PGC1α and SIRT1, regulate energy metabolism and prevent physiological fatigue in the muscles [35]. Therefore, to maintain skeletal muscle mass, quality, and strength, these myogenic gene expression and signals must be harmoniously regulated in myoblasts. However, their imbalanced regulation can cause sarcopenia.

In the present study, we selected MuRF1 expression-suppressing and AKT activation-inducing Lp and Bb, which belonged to *L. paracasei* and *Bifidobacterium bifidum*, respectively, in dexamethasone-stimulated C2C12 cells from human fecal lactic acid bacteria collection. They significantly increased muscle weight, including GA muscle, and physical strength and endurance in aged mice. They also increased AKT and mTOR activation and MyHC expression in GA muscle, while FOXO3a and NF-κB activation decreased. The activation of FOXO3a and NF-κB increases MuRF1 and MAFbx/atrogin-1 expression and decreases MyHC expression [33, 34]. These results suggest that these probiotics may decrease muscle protein degradation-inducing MuRF1 and MAFbx/atrogin-1 expression and increase differentiation/proliferation-inducing MyHC expression through the suppression of FOXO3a and NF-κB activation, resulting in the alleviation of ageing-dependent muscle weight loss and strength and endurance weakness.

Aging causes myofiber death from the accumulation of damaged mitochondria and weakens the intensity of physical exercise [36]. Oral administration of Lp, Bb, or LB increased PGC1α and SIRT1 expression in aged mice, while mtDNA expression increased in mice treated with LB alone. They increased running time and distance in the treadmill test. In addition, Chen et al. reported that *Lactobacillus casei*-contained probiotic supplementation attenuated age-related inflammation and reactive oxygen species production in SAMP8 mice by regulating gut microbiota and mitochondrial function [13]. These suggest that Lp and Bb, in particular, LB can alleviate ageing-dependent muscle fatigue by regulating mitochondrial function.

Aging is closely associated with chronic, low-grade inflammation [37, 38]. This inflammation increases the expression of proinflammatory cytokines such as TNF-α and IL-6 in muscle, brain, and intestine. These cytokines suppress protein synthesis and muscle proliferation and differentiation in the myoblast and BDNF expression in the neuronal cells [37, 39]. However, oral administration of Lp, Bb, or LB alleviated ageing-dependent expression of inflammatory markers TNF-α and IL-6 in the hippocampus, while IL-10 and BDNF expression increased. Furthermore, they suppressed FOXO3a activation and NF-κB-positive immune cell populations in hippocampus, while the BDNF-positive neuron cell population increased. In particular, Lp and LB alleviated ageing-dependent cognitive impairment-like behaviors in mice. They suppressed ageing-dependent LPS and corticosterone expression in the blood. LPS and corticosterone down-regulate BDNF expression [18, 40]. BDNF suppresses FOXO3 activation and neuronal differentiation [41, 42]. Kim et al. also reported that *Bifidobacterium longum*-contained probiotic supplementation alleviated mental flexibility with gut microbiota modulation in healthy older adults [43]. These results suggest that Lp and Bb, in particular LB, can alleviate ageing-dependent cognitive decline with neuroinflammation by increasing NF-κB-suppressed BDNF expression and decreasing BDNF-suppressed FOXO3a activation.

Oral administration of Lp, Bb, or LB shifted the β-diversity (PCoA analysis) of gut microbiota composition in aged mice, while the α-diversity (Shannon index) was not affected. At the family level, they also increased Prevotellaceae, Akkermandiaceae, and Bacteroidaceae populations, which were positively correlated with muscle weight and/or MyHC expression, and decreased Odoribacteraceae, Deferribacteraceae, Coriobacteriaceae, and Acholeplasmataceae populations, which were positively correlated with MuRF1 expression and/or IL-6 or TNF-α to IL-10 expression ratio. These results suggest that Odoribacteriaceae, Deferribacteraceae and Acholeplasmataceae may be inflammation-inducible and Akkermansiaceae and Bacteroidaceae may be inflammation-suppressible. These gut bacteria may control muscle protein synthesis and degradation-related transcription factors by regulating inflammation-related cytokine expression.

## Conclusions

Gut dysbiosis inducers such as ageing can cause systemic inflammation including colitis. Gut bacteria including inflammation-inducible Odoribacteriaceae, Deferribacteraceae, and Acholeplasmataceae and inflammation-suppressible Akkermansiaceae and Bacteroidaceae may be closely connected with muscle weight and strength by regulating muscle protein biosynthesis-related MyHC/MyoG and muscle degradation-related MuRF1/MAFbx expression and cognitive impairment by regulating hippocampal BDNF expression. Lp, Bb, and LB, can alleviate muscle weight loss and atrophy (strength and endurance) cognitive impairment by regulating gut microbiota-mediated AKT, NF-κB, and/or FOXO3a signaling pathways.

### Supplementary Information

**Additional file 1:** The present manuscript contains supplementary materials. **Table S1.** Primers for qPCR. **Table S2.** Whole genome properties of P61 and P62. **Table S3.** Effects of Bb, Lp, and LB on the gut microbiota composition at the phylum level. **Table S4.** Effects of Bb, Lp, and LB on the gut microbiota composition at the family level. **Table S5.** Effects of Bb, Lp, and LB on the gut microbiota composition at the genus level. **Table S5.** Effects of Bb, Lp, and LB on the gut microbiota composition at the species level. **Figure S1.** Effects of Bb, Lp, and LB on LPS-induced NF-κB activation in C2C12 cells. **Figure S2.** Effects of Bb, Lp, and their (4:1, 1:1, and 1:4) mix LB on LPS-induced IL-6 expression in C2C12 cells. **Figure S3.** Effects of Bb, Lp, and LB on bodyweights in aged mice. **Figure S4.** Effects of Bb, Lp, and LB on pAKT (a), AKT (b), p-mTOR (c), mTOR (d), and β-actin (e) in the GA muscle of aged mice. **Figure S5.** Effects of Bb, Lp, and LB on p-p65 (a), p65 (b), p16 (c), p-Foxo3a (d), Foxo3a (e), MuRF1 (f), MAFbx (g), PGC1a (h), MyHC (i), and β-actin (j) in the GA muscle of aged mice.

## Data Availability

Gut microbiota sequence data were deposited in the NCBI (PRJNA1011499). Other datasets used and/or analyzed during the current study are available from the corresponding author on request.

## Abbreviations

Bb: *Bifidobacterium bifidum *P61
BDNF: Brain-derived neurotropic factor
EDL: Extensor digitorum longus
ELISA: Enzyme-linked immunosorbent assay
GA: Gastrocnemius
H&E: Hematoxylin and eosin
IL: Interleukin
Lp: *Lactobacillus paracasei *P62
LPS: Lipopolysaccharide
MAFbx: Muscle atrophy F-box gene
MuRF1: Muscle RING-finger protein-1
mTor: Mammalian target of rapamycin
MyHC: Myosin heavy chain
PGC: Peroxisome proliferator-activated receptor gamma coactivator
QD: Quadriceps femoris
qPCR: Q*uantitative real-time polymerase chain reaction*
SOL: Soleus
TNF: Tumor necrosis factor
TA: Tibialis anterior
