# Supplementary material for: Bifidobacterium bifidum and Lactobacillus paracasei alleviate sarcopenia and cognitive impairment in aged mice by regulating gut microbiota-mediated AKT, NF-κB, and FOXO3a signaling pathways
Source: Immun Ageing. 2023 Oct 23;20:56. doi: 10.1186/s12979-023-00381-5 (PMC10591382; doi:10.1186/s12979-023-00381-5)
Supplement: Supplementary file 1 — Additional file 1: The present manuscript contains supplementary materials. Table S1. Primers for qPCR. Table S2. Whole genome properties of P61 and P62. Table S3. Effects of Bb, Lp, and LB on the gut microbiota composition at the phylum level. Table S4. Effects of Bb, Lp, and LB on the gut microbiota composition at the family level. Table S5. Effects of Bb, Lp, and LB on the gut microbiota composition at the genus level. Table S5. Effects of Bb, Lp, and LB on the gut microbiota composition at the species level. Figure S1. Effects of Bb, Lp, and LB on LPS-induced NF-κB activation in C2C12 cells. Figure S2. Effects of Bb, Lp, and their (4:1, 1:1, and 1:4) mix LB on LPS-induced IL-6 expression in C2C12 cells. Figure S3. Effects of Bb, Lp, and LB on bodyweights in aged mice. Figure S4. Effects of Bb, Lp, and LB on pAKT (a), AKT (b), p-mTOR (c), mTOR (d), and β-actin (e) in the GA muscle of aged mice. Figure S5. Effects of Bb, Lp, and LB on p-p65 (a), p65 (b), p16 (c), p-Foxo3a (d), Foxo3a (e), MuRF1 (f), MAFbx (g), PGC1a (h), MyHC (i), and β-actin (j) in the GA muscle of aged mice. [file 12979_2023_381_MOESM1_ESM.docx]

[Supplement]

[Immunity and Aging]

***Bifidobacterium bifidum* and *Lactobacillus paracasei* alleviate sarcopenia and cognitive impairment in aged mice** **by regulating gut microbiota-mediated AKT, NF-κB, and FOXO3a signaling pathways**

**Table S1**. Primers for qPCR

| Gene | Primer | |
| --- | --- | --- |
|  | Forward | Reverse |
| MuRF1 | 5′-AGCCAAGACAATAGAGATGCCTACTTC-3′ | 5′-GGCCTTGAACTCATAGAGATCCAAC-3′ |
| MAFbx/  Atrogin-1 | 5′-AAGGAAGATGAACGCTGTCA-3′ | 5′-ATTGCCTCCCAGATAAAGTATGT-3′ |
| TNF-α | 5′-GATTATGGCTCAGGGTCCAA-3′ | 5′-GCTCCAGTGAATTCGGAAAG-3′ |
| IL-6 | 5′-TAGTCCTTCCTACCCCAATTTCC-3’ | 5′-TTGGTCCTTAGCCACTCCTTC-3’ |
| MyHC | 5′-ACAAGCTGCGGGTGAAGAGC-3′ | 5′-CAGGACAGTGACAAAGAACG-3′ |
| MyHC-2A | 5′-AAGCGAAGAGTAAGGCTGTC-3′ | 5′-GTGATTGCTTGCAAAGGAAC-3′ |
| MyHC-2X | 5′-CACCGTCTGGATGAGGCTGA-3′ | 5′-TGTTTGCGCAGACCCTTGATAG-3′ |
| MyHC-2B | 5′-GATTGACGTGGAGAGGTCTAAC-3′ | 5′-CCTGAGTTTCCTCGTACTTCTG-3′ |
| MyoG | 5′-GGCTGCCTAAAGTGGAGATCCT-3′ | 5′-AGGCCTGTAGGCGCTCAAT-3′ |
| PGC-1α | 5′-TGATGTGAATGACTTGGATACAGACA-3’ | 5′-GCTCATTGTTGTACTGGTTGGATATG-3′ |
| SIRT1 | 5′-AGGGAACCTTTGCCTCATCTAC-3’ | 5′-GGTGGCAACTCTGATAAATGAAC-3′ |
| mtDNA | 5′-TTTTATCTGCATCTGAGTTTAA-3’ | 5’-CCACTTCATCTTACCATTTAT-3’ |
| Beta-actin | 5′-CCATCCTGCGTCTGGACCTG-3′ | 5′-CTCGTCATACTCCTGCTTGC-3′ |

Table S2. Whole genome properties of P61 and P62

| Strain | P61 | P62 |
| --- | --- | --- |
| Taxonomy | *Bifidobacterium bifidum* | *Lacticaseibacillus paracasei* |
| Assembly type | COMPLETE | contigs |
| Contig | 1 | 9 |
| Genome size (bp) | 2,194,882 | 3,165,307 |
| N50 (bp) | 2,194,882 | 2,887,200 |
| CDS | 1,773 | 3,133 |
| Mean of CDS length (bp) | 1,057.6 (844.2) | 855.1 (604.8) |
| Median of CDS length (bp) | 882 | 750 |
| GC rato (%) | 62.5% | 46.3% |
| No. of rRNA genes | 9 | 15 |
| No. of tRNA genes | 52 | 63 |
| Phylogenetic tree of P61  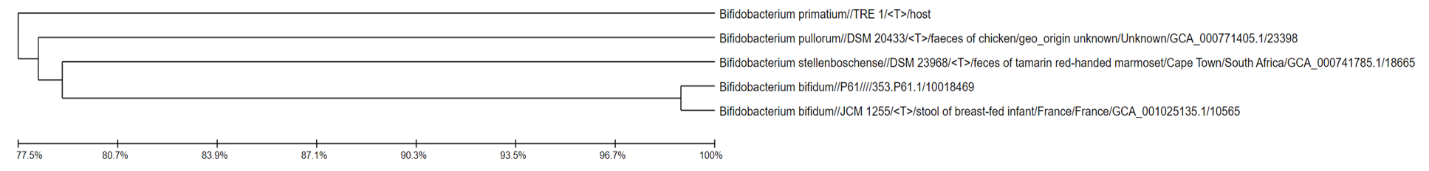 | | |
| Phylogenetic tree of P62  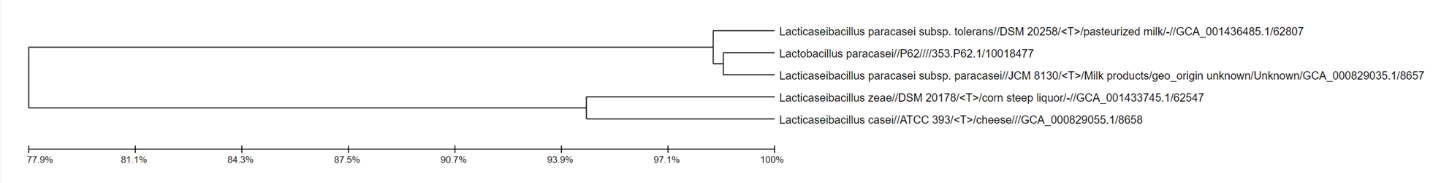 | | |

Table S3. Effects of Bb, Lp, and LB on the gut microbiota composition at the phylum level

| Taxon Name | Composition (%)^1^ | | | | |
| --- | --- | --- | --- | --- | --- |
|  | Vh | Bb | Lp | LB | Cr |
| Bacteroidetes | 49.4 ± 6.5 | 54.4 ± 9.7 | 50.2 ± 5.4 | 57.0 ± 2.1***** | 61.2 ± 7.6***** |
| Firmicutes | 43.4 ± 9.0 | 35.5 ± 6.3 | 45.2 ± 5.1 | 27.5 ± 10.1***** | 27.7 ± 8.8***** |
| Proteobacteria | 5.3 ± 2.8 | 8.5 ± 4.8 | 2.7 ± 1.1 | 8.0 ± 4.9 | 7.2 ± 2.1 |
| Tenericutes | 0.5 ± 0.2 | 0.6 ± 0.5 | 0.8 ± 0.9 | 0.3 ± 0.2 | 0.5 ± 0.3 |
| Deferribacteres | 0.8 ± 0.7 | 0.0 ± 0.1***** | 0.5 ± 0.4 | 0.0 ± 0.0***** | 0.0 ± 0.0***** |
| Actinobacteria | 0.3 ± 0.2 | 0.2 ± 0.1 | 0.2 ± 0.1 | 0.2 ± 0.1 | 0.6 ± 0.5 |
| Cyanobacteria | 0.1 ± 0.2 | 0.7 ± 0.3***** | 0.1 ± 0.1 | 0.5 ± 0.9 | 0.5 ± 0.9 |
| Verrucomicrobia | 0.2 ± 0.3 | 0.1 ± 0.0 | 0.2 ± 0.5 | 6.4 ± 6.6***** | 2.1 ± 3.1 |
| Saccharibacteria_TM7 | 0.0 ± 0.0 | 0.1 ± 0.0***** | 0.0 ± 0.0 | 0.0 ± 0.0 | 0.0 ± 0.0 |

^1)^ Mean ± SD. *p<0.05 vs. Vh.

Table S4. Effects of Bb, Lp, and LB on the gut microbiota composition at the family level

| Taxon Name | Composition (%)^1^ | | | | |
| --- | --- | --- | --- | --- | --- |
|  | Vh | Bb | Lp | LB | Cr |
| Muribaculaceae | 39.5±7.9 | 38.0±7.2 | 35.1±7.3 | 33.9±7.3 | 42.2±5.5 |
| Lachnospiraceae | 31.5±8.9 | 23.1±7.1 | 33.2±4.4 | 15.6±8.5***** | 17.5±8.8***** |
| Ruminococcaceae | 7.8±2.5 | 6.0±1.4 | 8.4±2.8 | 5.6±3.6 | 4.2±1.7***** |
| Prevotellaceae | 3.0±2.8 | 6.1±1.6***** | 5.0±2.5 | 6.4±3.6 | 8.3±4.5***** |
| Rikenellaceae | 3.0±2.1 | 3.7±1.6 | 3.4±1.6 | 5.0±2.2 | 2.3±1.0 |
| Bacteroidaceae | 2.2±0.9 | 3.1±1.4 | 5.2±3.9 | 9.0±5.8***** | 6.3±5.8 |
| Desulfovibrionaceae | 1.4±1.1 | 2.7±1.6 | 1.4±0.6 | 1.6±0.7 | 2.0±1.5 |
| Erysipelotrichaceae | 1.5±1.6 | 1.0±1.1 | 0.3±0.2 | 2.3±1.5 | 3.4±2.6 |
| Sutterellaceae | 1.3±1.4 | 1.0±0.6 | 0.5±0.3 | 2.6±2.1 | 2.8±1.1 |
| Pseudomonadaceae | 1.5±2.0 | 0.3±0.2 | 0.2±0.1 | 1.7±2.5 | 1.3±2.2 |
| Lactobacillaceae | 1.0±0.6 | 2.5±3.1 | 1.3±1.0 | 3.0±1.2 | 0.9±0.5 |
| Christensenellaceae | 0.8±0.5 | 2.3±1.2 | 1.4±1.1 | 0.5±0.3 | 0.8±0.4 |
| Odoribacteraceae | 0.9±0.4 | 1.1±0.6 | 0.7±0.9 | 0.5±0.4 | 0.9±0.6 |
| Helicobacteraceae | 0.7±0.2 | 3.8±4.4 | 0.4±.0.0***** | 0.8±1.1 | 0.5±0.2 |
| Deferribacteraceae | 0.8±0.7 | 0.0±0.1***** | 0.5±0.4 | 0.0±0.0***** | 0.0±0.0***** |
| AC160630_f | 0.5±0.4 | 1.7±1.1 | 0.4±0.4 | 0.3±0.2 | 0.4±0.2 |
| Acholeplasmataceae | 0.3±0.2 | 0.2±0.2 | 0.6±0.7 | 0.0±0.0***** | 0.2±0.2 |
| Rhodospirillaceae | 0.3±0.4 | 0.6±0.4 | 0.2±0.1 | 0.4±0.3 | 0.4±0.3 |
| Dehalobacterium_f | 0.2±0.1 | 0.3±0.2 | 0.4±0.1 | 0.2±0.1 | 0.2±0.2 |
| Porphyromonadaceae | 0.2±0.2 | 0.8±0.4 | 0.3±0.2 | 1.6±2.4 | 0.6±0.5 |
| Peptostreptococcaceae | 0.3±0.5 | 0.0±0.1 | 0.0±0.0 | 0.2±0.2 | 0.4±0.3 |
| PAC000197_f | 0.1±0.1 | 0.2±0.2 | 0.1±0.1 | 0.1±0.1 | 0.1±0.1 |
| Bifidobacteriaceae | 0.2±0.1 | 0.1±0.1 | 0.1±0.1 | 0.1±0.1 | 0.4±0.4 |
| FR888536_f | 0.1±0.2 | 0.7±0.3 | 0.1±0.1 | 0.5±0.9 | 0.5±0.9 |
| Coriobacteriaceae | 0.2±0.1 | 0.1±0.0 | 0.1±0.0 | 0.1±0.1 | 0.2±0.1 |
| Akkermansiaceae | 0.2±0.3 | 0.1±0.0 | 0.2±0.5 | 6.4±6.6***** | 2.1±3.1 |

^1)^ Mean ± SD. *p<0.05 vs. Vh.

Table S5. Effects of Bb, Lp, and LB on the gut microbiota composition at the genus level

|  | Composition (%)^1^ | | | | |
| --- | --- | --- | --- | --- | --- |
|  | Vh | Bb | Lp | LB | Cr |
| PAC001068_g | 10.9±4.6 | 16.2±4.0 | 10.1±3.5 | 14.6±2.5 | 10.9±2.9 |
| PAC000664_g | 6.6±2.0 | 2.4±1.5***** | 5.4±3.6 | 2.4±2.1***** | 4.0±3.2 |
| PAC000186_g | 6.6±2.8 | 6.7±1.2 | 4.0±0.8 | 4.5±1.9 | 6.7±2.0 |
| PAC001112_g | 4.2±4.1 | 2.9±1.9 | 6.5±4.6 | 2.6±2.2 | 6.0±2.9 |
| Muribaculum | 4.6±1.2 | 3.8±1.3 | 5.5±3.3 | 2.5±0.8***** | 6.3±3.9 |
| PAC000198_g | 4.1±1.2 | 3.0±1.2 | 3.4±1.2 | 3.4±0.5 | 3.5±1.2 |
| Oscillibacter | 3.6±2.2 | 1.9±0.8 | 3.4±1.8 | 1.2±1.0***** | 1.2±0.7***** |
| KE159538_g | 4.0±4.1 | 5.3±2.1 | 6.8±8.8 | 2.1±4.0 | 1.2±0.7 |
| Alistipes | 2.9±2.2 | 3.3±1.7 | 2.7±1.3 | 4.3±2.3 | 1.7±0.8 |
| Pseudoflavonifractor | 2.2±0.9 | 1.9±0.7 | 2.7±0.4 | 1.9±1.8 | 1.6±0.8 |
| LLKB_g | 2.5±1.6 | 1.2±1.0 | 3.1±3.5 | 0.9±1.0 | 0.7±0.7***** |
| Bacteroides | 2.2±0.9 | 3.1±1.4 | 5.2±3.9 | 9.0±5.8***** | 6.3±5.8 |
| PAC001092_g | 1.8±1.5 | 1.1±0.8 | 2.4±1.3 | 0.8±0.7 | 1.8±1.6 |
| PAC001063_g | 2.0±1.4 | 0.7±0.5***** | 1.4±0.6 | 0.5±0.5***** | 1.3±0.9 |
| Prevotellaceae_uc | 1.6±1.8 | 3.4±2.2 | 3.1±2.0 | 4.3±2.7 | 4.7±3.7 |
| Prevotella | 1.2±1.0 | 1.5±0.6 | 1.7±0.7 | 1.3±1.0 | 2.4±1.1 |
| Eubacterium_g6 | 1.1±1.1 | 1.1±0.9 | 1.7±1.3 | 0.4±0.5 | 0.2±0.1 |
| KE159600_g | 0.9±1.3 | 1.0±0.9 | 1.7±1.6 | 0.4±0.6 | 1.2±1.2 |
| PAC002400_g | 1.5±1.2 | 0.9±0.3 | 0.6±0.5 | 0.5±0.6 | 1.9±1.9 |
| PAC001127_g | 1.4±1.2 | 0.6±0.5 | 0.8±0.4 | 0.4±0.3 | 0.9±0.8 |
| KE159571_g | 1.3±1.0 | 0.6±0.6 | 1.3±0.6 | 0.6±0.4 | 1.0±0.9 |
| Pseudomonas | 1.5±2.0 | 0.3±0.2 | 0.2±0.1 | 1.7±2.5 | 1.3±2.2 |
| PAC001066_g | 1.3±0.7 | 0.4±0.2 | 0.7±0.2 | 0.9±0.5 | 1.3±0.4 |
| Anaerotignum | 1.1±1.3 | 0.8±0.5 | 0.8±0.2 | 0.4±0.4 | 0.5±0.3 |
| Turicimonas | 1.0±1.5 | 0.9±0.5 | 0.3±0.3 | 2.3±2.1 | 1.8±1.2 |
| Akkermansia | 0.2±0.3 | 0.1±0.0 | 0.2±0.5 | 6.4±6.6***** | 2.1±3.1 |

^1)^ Mean ± SD. *p<0.05 vs. Vh.

Table S6. Effects of Bb, Lp, and LB on the gut microbiota composition at the species level

|  | Composition (%)^1^ | | | | |
| --- | --- | --- | --- | --- | --- |
|  | Vh | Bb | Lp | LB | Cr |
| PAC002450_s | 3.4±4.3 | 2.7±1.7 | 1.6±2.0 | 2.2±0.7 | 2.7±3.1 |
| PAC001515_s | 3.2±1.8 | 1.5±1.4 | 4.0±3.0 | 0.9±1.4***** | 2.8±2.5 |
| EF097112_s | 3.0±1.9 | 1.3±0.9 | 3.5±2.0 | 2.4±0.9 | 2.6±1.8 |
| PAC001065_s group | 3.2±1.7 | 0.7±0.8***** | 2.0±0.8 | 0.4±0.4***** | 2.1±2.0 |
| PAC001112_s | 1.9±3.0 | 1.1±1.5 | 3.5±3.5 | 0.9±1.2 | 2.5±2.7 |
| PAC001064_s | 2.3±1.6 | 1.3±0.5 | 0.8±0.3 | 1.3±0.6 | 2.8±1.6 |
| PAC001063_s group | 2.0±1.4 | 0.6±0.5***** | 1.4±0.6 | 0.5±0.5***** | 1.3±0.9 |
| PAC000198_s | 1.7±1.1 | 1.5±1.1 | 1.5±0.6 | 1.3±0.8 | 1.3±0.9 |
| PAC002481_s | 1.2±1.0 | 1.5±0.6 | 1.6±0.7 | 1.3±1.0 | 2.4±1.1 |
| Muribaculum intestinale | 1.8±1.3 | 2.2±0.8 | 0.9±0.3 | 1.9±0.8 | 1.6±1.0 |
| PAC001770_s | 1.9±3.5 | 0.0±0.0 | 0.0±0.0 | 0.1±0.0 | 0.3±0.5 |
| PAC001084_s | 1.4±1.4 | 0.1±0.1***** | 0.3±0.4 | 0.9±0.6 | 0.4±0.6 |
| PAC002400_s | 1.5±1.2 | 0.9±0.3 | 0.5±0.5 | 0.5±0.6 | 1.9±1.9 |
| PAC002399_s | 1.4±1.2 | 0.6±0.5 | 0.8±0.4 | 0.4±0.3 | 0.9±0.5 |
| PAC001488_s group | 1.5±1.8 | 0.0±0.0 | 0.0±0.0 | 0.2±0.6 | 0.0±0.0 |
| PAC002530_s group | 1.2±0.9 | 0.9±0.8 | 1.5±1.2 | 0.4±0.5 | 1.4±0.8 |
| PAC001077_s | 1.5±1.5 | 0.7±0.7 | 3.0±4.1 | 0.2±0.3 | 3.1±3.8 |
| PAC001092_s | 1.0±1.6 | 0.5±0.6 | 1.6±1.6 | 0.4±0.7 | 1.5±1.6 |
| PAC001139_s | 1.3±0.3 | 0.5±0.4 | 1.1±0.5 | 0.4±0.3***** | 1.4±1.1 |
| PAC001066_s | 1.3±0.7 | 0.4±0.2***** | 0.7±0.2 | 0.9±0.5 | 1.3±0.4 |
| PAC001072_s | 1.2±0.6 | 1.3±0.4 | 0.9±0.3 | 0.9±0.3 | 1.7±1.4 |
| PAC001383_s | 1.3±1.1 | 0.3±0.4 | 2.0±2.8 | 0.3±0.4 | 0.4±0.4 |
| PAC001070_s group | 0.4±.3 | 10.0±4.8 | 2.3±2.7 | 7.3±1.9***** | 1.7±2.6 |
| PAC002444_s | 0.9±1.5 | 0.9±1.0 | 1.1±1.3 | 0.9±1.3 | 0.2±0.2 |
| KE159538_s | 1.1±1.2 | 0.7±1.1 | 0.3±0.4 | 0.2±0.2 | 0.3±0.3 |
| Akkermansia muciniphila | 0.2±0.3 | 0.1±0.0 | 0.2±0.5 | 6.4±6.6***** | 2.1±3.1 |

^1)^ Mean ± SD. *p<0.05 vs. Vh.

**(a)**


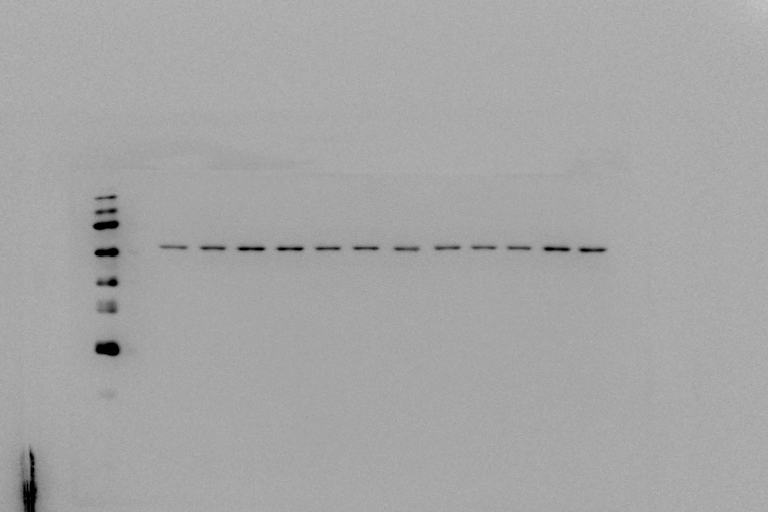


**95**

**56**

**28**

**17**

**35**

**70**

**43**

**kDa**

**130**

**LPS**

**NC**

**Vh**

**Lp**

**Bb**

**LB**

**Cr**

**65kDa**

**p-NF-kB p65**

(b)


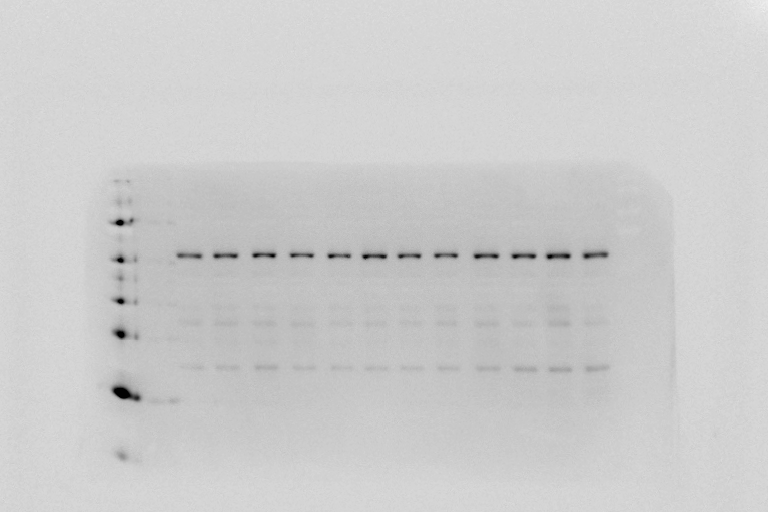


**95**

**56**

**28**

**17**

**35**

**70**

**43**

**kDa**

**130**

**65kDa**

**LPS**

**NC**

**Vh**

**Lp**

**Bb**

**LB**

**Cr**

**NF-kB p65**

(c)


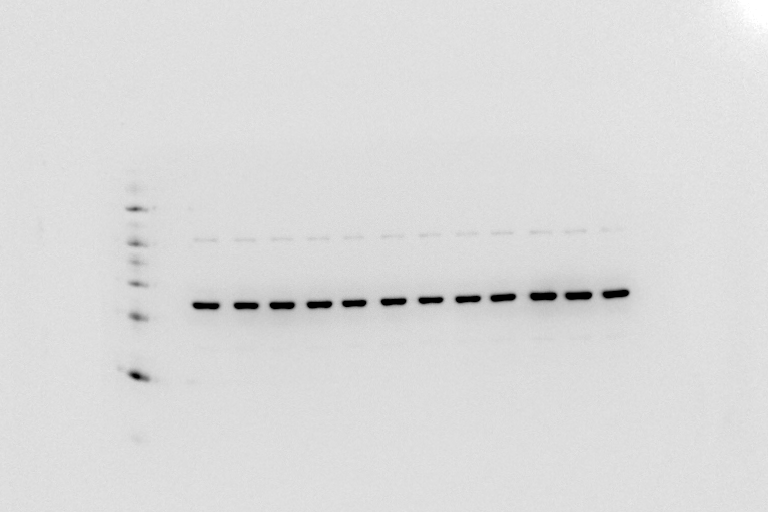


**35**

**43**

**56**

**90**

**kDa**

**70**

**42kDa**

**Beta actin**

**LPS**

**NC**

**Vh**

**Lp**

**Bb**

**LB**

**Cr**

Figure S1. Effects of Bb, Lp, and LB on LPS-induced NF-κB activation in C2C12 cells. Effects on p-p65 (a) and p65 (b), and β-actin expression (c).

Figure S2. Effects of Bb, Lp, and their (4:1, 1:1, and 1:4) mix LB on LPS-induced IL-6 expression in C2C12 cells. C2C12 cells (1 × 10^5^ cells/mL) were treated with LPS (100 ng/mL) in the absence or presence of Bb, alone Lp (1 × 10^4^ or 1 × 10^6^ colon-forming units [CFUs] /mL), or LB (1 × 10^6^ CFUs /mL). Data are indicated as mean ± SD (n = 4). ^#^p<0.05 vs NC. ^*^p<0.05 vs. group treated with vehicle with LPS.

Figure S3. Effects of Bb, Lp, and LB on bodyweights in aged mice. Data are indicated as mean ± SD (n = 7).

**In vivo**

(a)

**
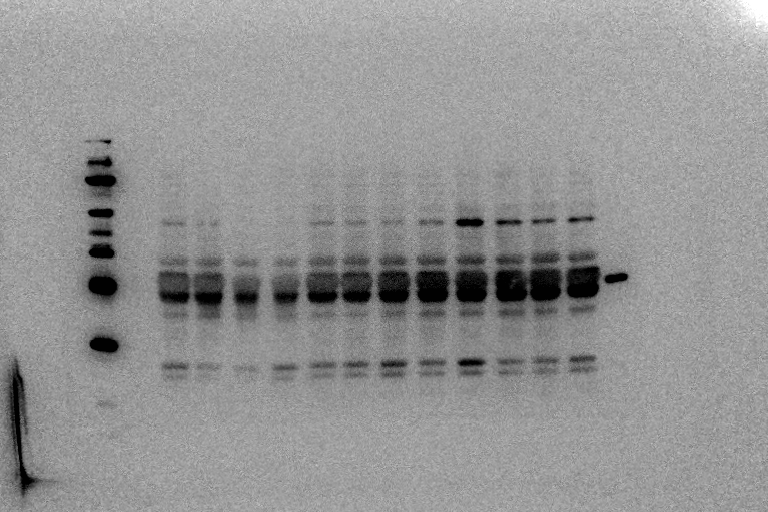
**

**kDa**

**Ag**

**Yg**

**Vh**

**Lp**

**Bb**

**LB**

**Cr**

**170**

**130**

**90**

**70**

**56**

**35**

**17**

**28**

**43**

**p-Akt**

**60kDa**

**70**

(b)

**
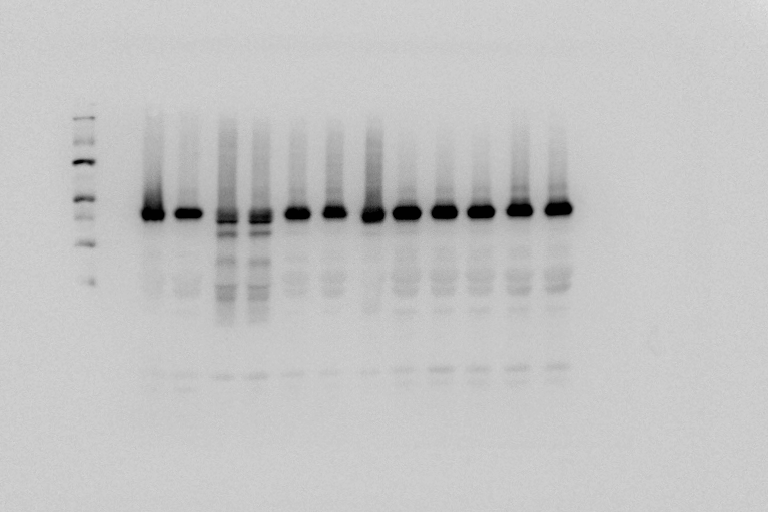
**

**Akt**

**70**

**kDa**

**Ag**

**Yg**

**Vh**

**Lp**

**Bb**

**LB**

**Cr**

**35**

**43**

**56**

**60kDa**

**170**

**130**

**90**

(c)

**p-mTOR**


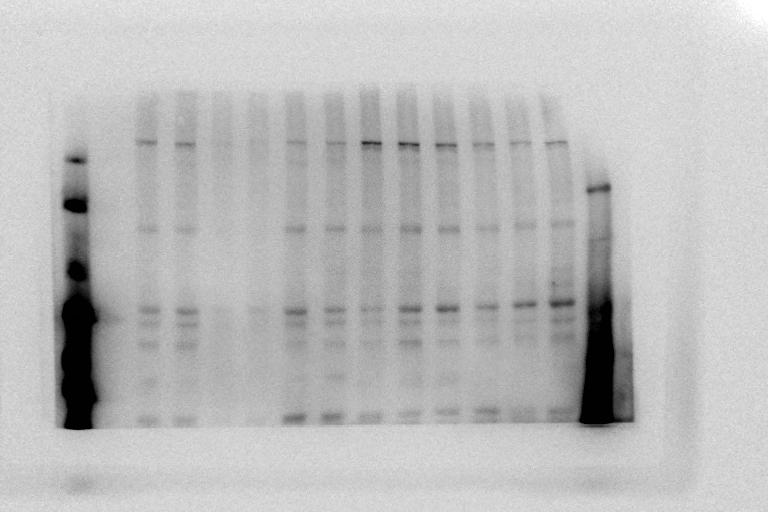


**kDa**

**Ag**

**Yg**

**Vh**

**Lp**

**Bb**

**LB**

**Cr**

**130**

**170**

**95**

**289 kD**

**70**

(d)


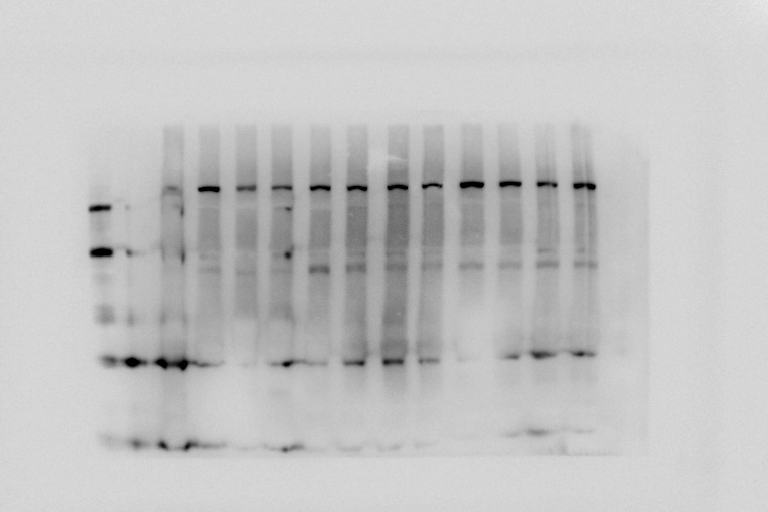


**mTOR**

**kDa**

**Ag**

**Yg**

**Vh**

**Lp**

**Bb**

**LB**

**Cr**

**289 kD**

**95**

**70**

**130**

**170**

(e)


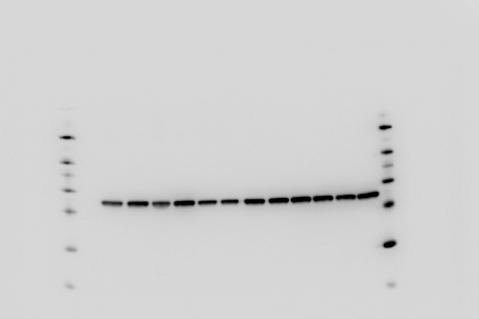


**43kD**

**43**

**Ag**

**Yg**

**Vh**

**Lp**

**Bb**

**LB**

**Cr**

**kDa**

**90**

**72**

**17**

**56**

**35**

**Beta-actin**

Figure S4. Effects of Bb, Lp, and LB on pAKT (a), AKT (b), p-mTOR (c), mTOR (d), and β-action (e) in the GA muscle of aged mice.

(a)

**p-NF-kB p65**

**
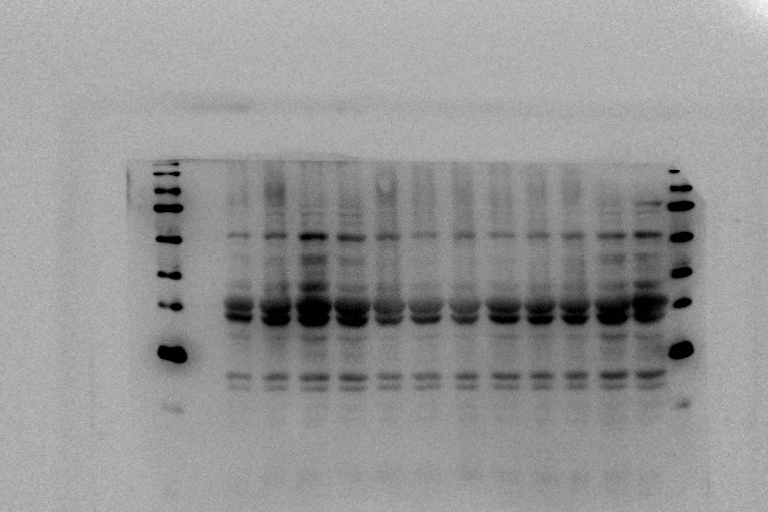
**

**Ag**

**Yg**

**Vh**

**Lp**

**Bb**

**LB**

**Cr**

**95**

**56**

**65kDa**

**28**

**17**

**35**

**70**

**43**

**130**

**kDa**

(b)


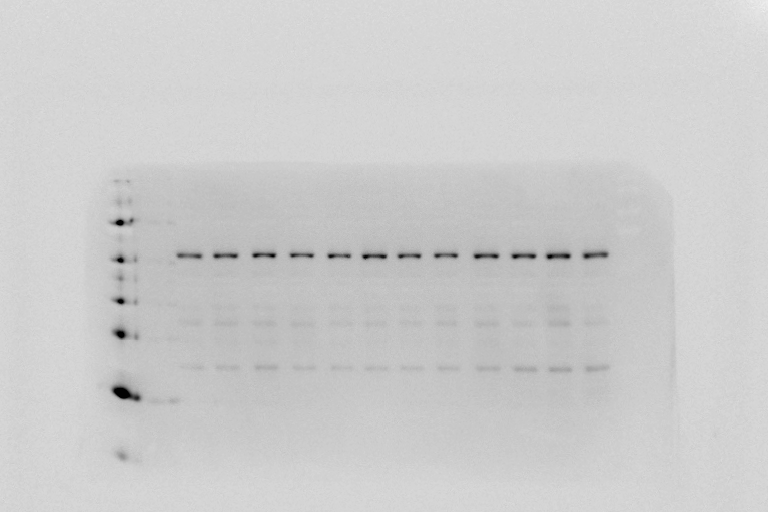


**56**

**Ag**

**Yg**

**Vh**

**Lp**

**Bb**

**LB**

**Cr**

**65kDa**

**kDa**

**130**

**95**

**70**

**35**

**43**

**28**

**17**

**NF-kB p65**

(c)

**Ag**

**Yg**

**Vh**

**Lp**

**Bb**

**LB**

**Cr**


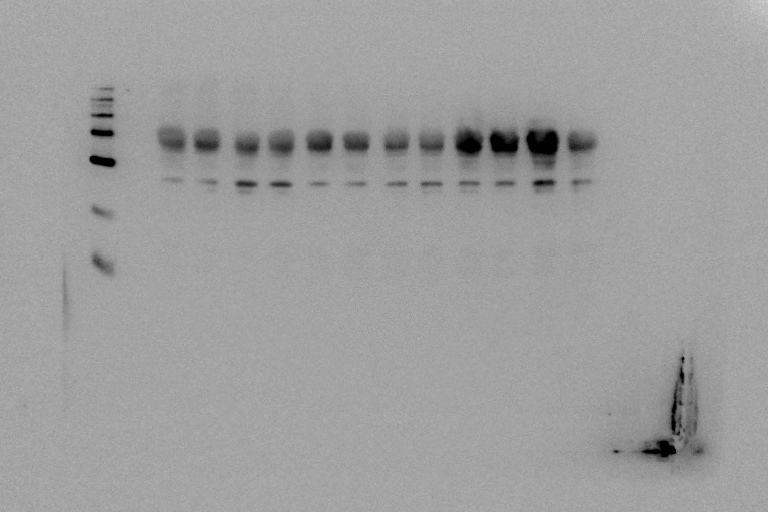


**56**

**10**

**28**

**35**

**43**

**kDa**

**P16**

**16kDa**

**17**

(d)


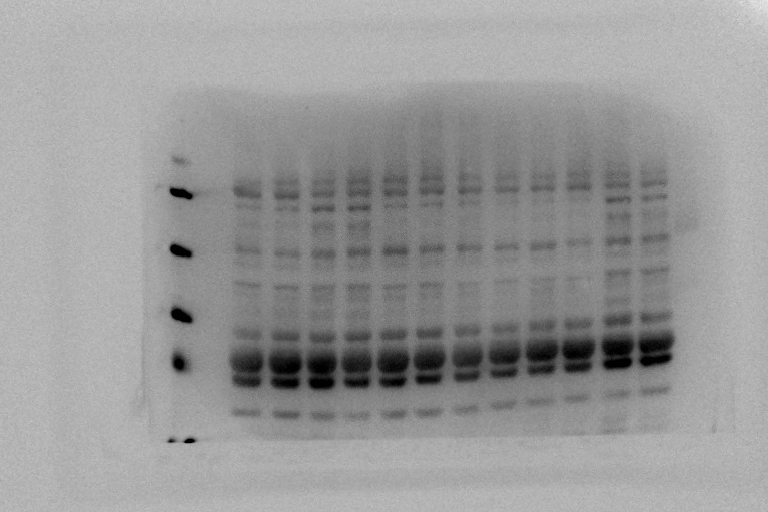


**p-foxO3a**

**Ag**

**Yg**

**Vh**

**Lp**

**Bb**

**LB**

**Cr**

**kDa**

**130**

**100**

**97 kD**

**43**

**56**

**70**

(e)

**foxO3a**


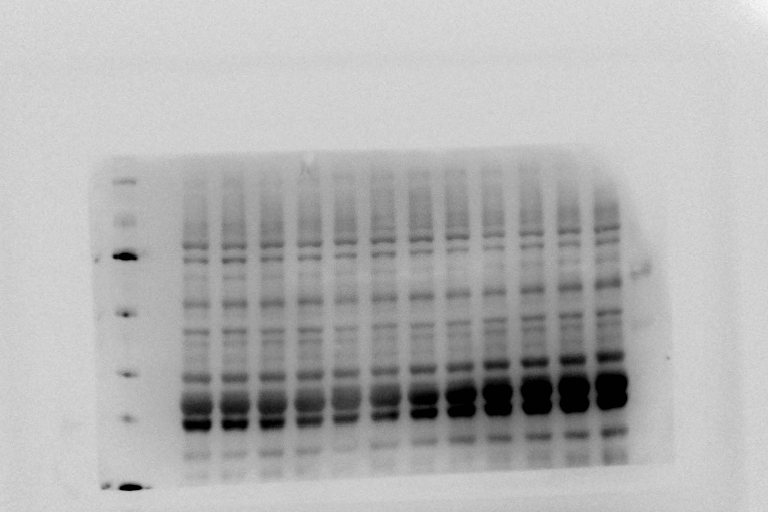


**Ag**

**Yg**

**Vh**

**Lp**

**Bb**

**LB**

**Cr**

**56**

**70**

**97 kD**

**100**

**130**

**kDa**

(f)


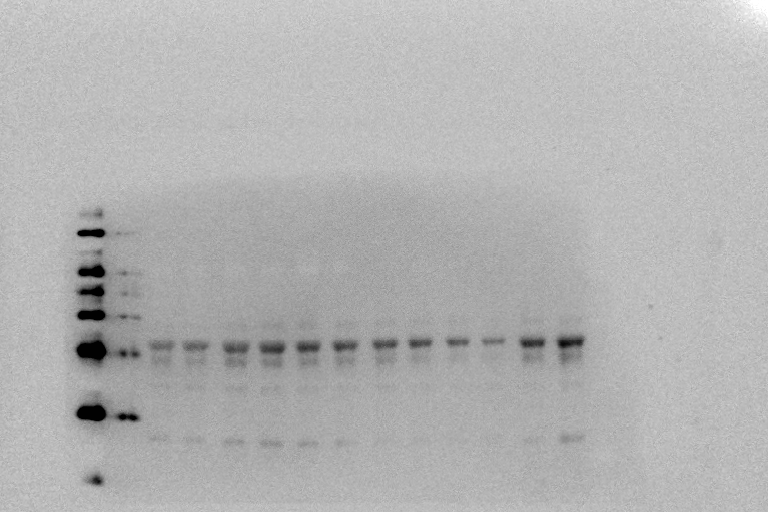


**Ag**

**Yg**

**Vh**

**Lp**

**Bb**

**LB**

**Cr**

**kDa**

**43 kDa**

**130**

**43**

**56**

**70**

**35**

**95**

**MuRF1**

(g)

**MAFbx-1/Atrogin1**


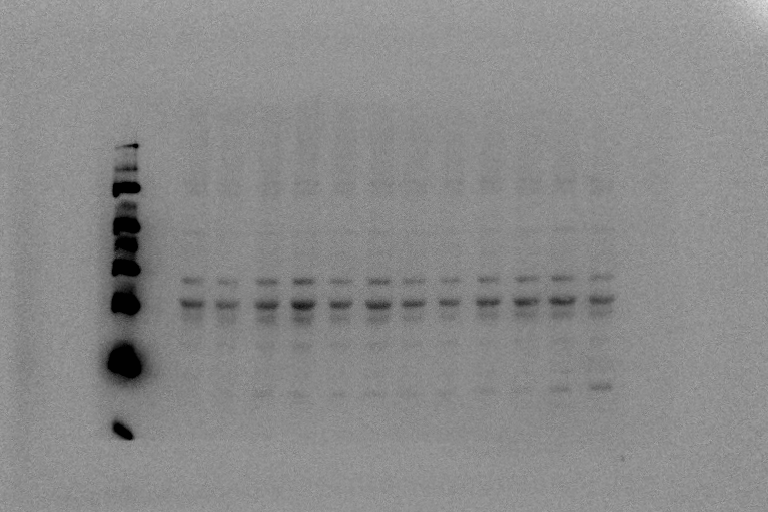


**Ag**

**Yg**

**Vh**

**Lp**

**Bb**

**LB**

**Cr**

**130**

**70**

**35**

**43**

**95**

**56**

**kDa**

**42 kDa**

(h)


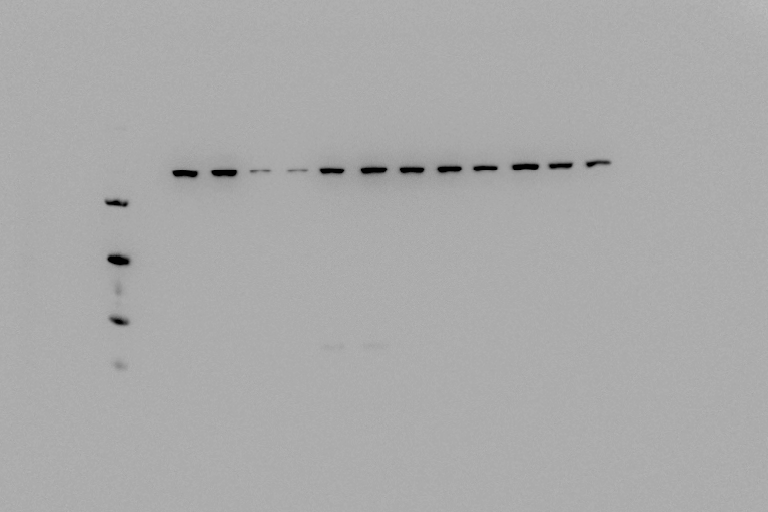


**PGC-1α**

**kDa**

**Ag**

**Yg**

**Vh**

**Lp**

**Bb**

**LB**

**Cr**

**35**

**43**

**56**

**70**

**90 kDa**

(i)


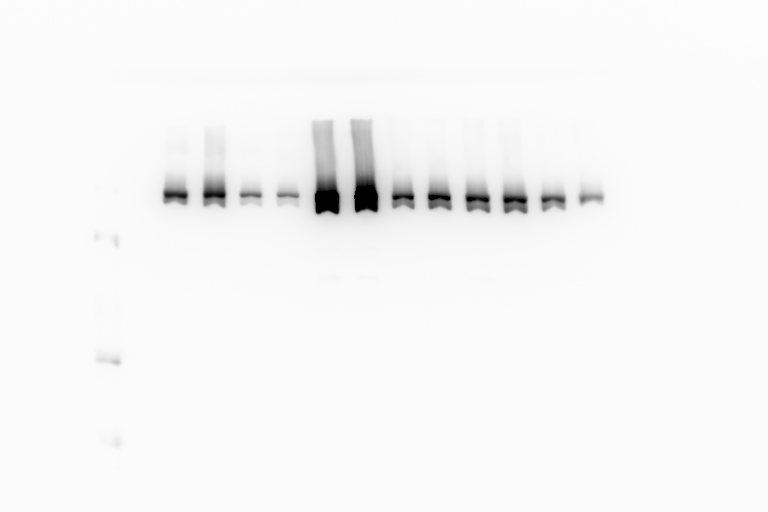


**Ag**

**Yg**

**Vh**

**Lp**

**Bb**

**LB**

**Cr**

**MyHC**

**kDa**

**72**

**90**

**289 kDa**

**130**

Figure S5. Effects of Bb, Lp, and LB on p-p65 (a), p65 (b), p16 (c), p-Foxo3a (d), Foxo3a (e), Murif1 (f), MAFbx (g), PGC1a (h), MuHC (i), and β-actin (j) in the GA muscle of aged mice.

(a)


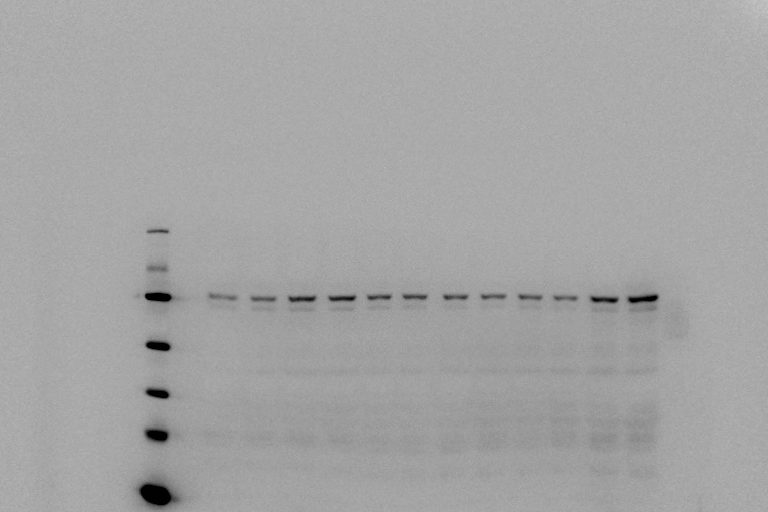


**97 kD**

**kDa**

**100**

**130**

**70**

**56**

**Ag**

**Yg**

**Vh**

**Lp**

**Bb**

**LB**

**Cr**

**p-FOXO3a**

(b)


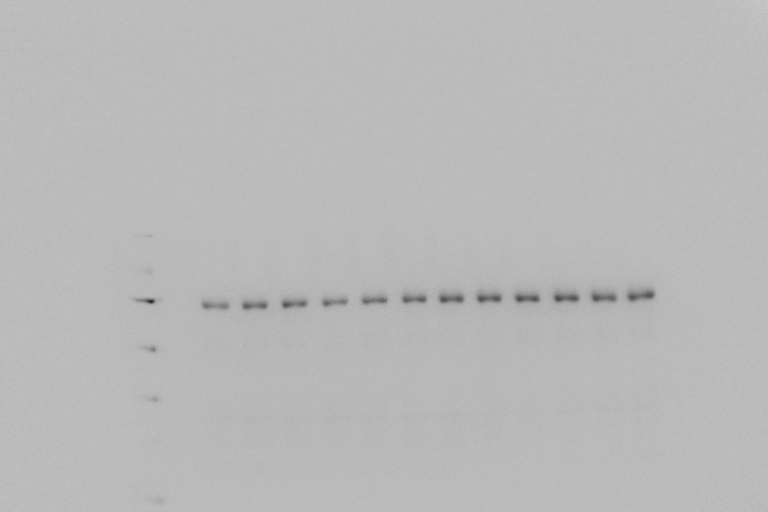


**FOXO3a**

**97 kD**

**kDa**

**100**

**130**

**70**

**56**

**Ag**

**Yg**

**Vh**

**Lp**

**Bb**

**LB**

**Cr**

Figure S6. Effects of Bb, Lp, and LB on the cognitive function in aged mice. (a) Effects on p-FOXO3a (a) and FOXO3a (b) expression, assessed by immunoblotting.
